# Factors associated with diet diversity among infants and young children in the Eastern and Southern Africa region

**DOI:** 10.1111/mcn.13487

**Published:** 2023-03-15

**Authors:** Yunhee Kang, Rebecca A. Heidkamp, Kudakwashe Mako‐Mushaninga, Aashima Garg, Joan N. Matji, Mara Nyawo, Hope C. Craig, Andrew L. Thorne‐Lyman

**Affiliations:** ^1^ Department of International Health Johns Hopkins Bloomberg School of Public Health Baltimore Maryland USA; ^2^ UNICEF Eastern and Southern Africa Regional Office Nairobi Kenya; ^3^ UNICEF Program Division New York City New York USA

**Keywords:** Eastern and Southern Africa, equity gap, infant and young children, minimum dietary diversity, risk factors

## Abstract

This study explores common factors associated with not meeting minimum dietary diversity (MDD) among 27,072 children aged 6–23 months in Eastern and Southern Africa using data from nine Demographic and Health Surveys from 2013 to 2016. MDD was defined as consumption of more than or equals to five of eight food groups including breast milk in the past 24 h. Equity gaps were calculated as the difference in MDD prevalence between the top and bottom wealth quintiles. Logistic regression was conducted to identify common factors for not meeting MDD at the household, maternal and child levels across two or more countries to inform regional policies to improve children's diets. Kenya had the highest MDD wealth equity gap (40.4 pts), and South Africa had the smallest (14.4 pts). Equity gaps for flesh foods or eggs (up to 39.8 pp) were larger than for grain or legumes (up to 20 pp). Common risk factors for not reaching MDD included younger child age (6–11 months) (*n* = 9 countries), no formal maternal occupation (*n* = 6), not receiving vitamin‐A supplementation (*n* = 3), younger maternal age (*n* = 3), lower maternal education (*n* = 3), no media (*n* = 3) or newspaper (*n* = 3) exposure, lower household wealth quintile (*n* = 3), use of nonefficient cooking fuel (*n* = 2), longer time to get to the water source (*n* = 2), not listening to the radio (*n* = 2) and higher birth order (*n* = 2). Priorities for improving MDD in the region include introducing diverse foods at a young age from 6 months with early nutrition counselling, promoting higher maternal education, increasing food purchasing power and ensuring the support of younger mothers.

## Key messages

- Diets of children in Eastern and Southern Africa (ESA) lack diversity.
- A number of common risk factors predict dietary diversity across countries.
- Risk factors for low dietary diversity include wealth, education and media access.
- Wide equity gaps were apparent for most food groups in ESA.
- Interventions addressing affordability and access to diverse diets are needed.

## Graphical abstract

Diets of children in Eastern and Southern Africa lack diversity with large equity gaps in most food groups. Risk factors for low dietary diversity include wealth, education and media access. Interventions addressing affordability and access to diverse diets are required.

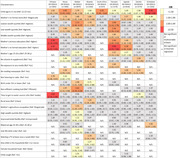

## 1 INTRODUCTION

The quality of diet during early childhood is a critical determinant of child's growth and development (Kuklina et al., 2004; Ruel, 2003; Miller et al., 2020; Prado et al., 2017). International guidelines recommend that children aged 6–23 months should continue to breastfeed and are introduced to age‐appropriate complementary foods from diverse food groups (World Health Organization, 2003). Dietary diversity, particularly the inclusion of foods rich in iron and zinc, is particularly important for this age group due to physiological requirements of rapid growth, depletion of stores acquired during gestation and small gastric capacity (Beluska‐Turkan et al., 2019; Dewey, 2013).

Minimum dietary diversity (MDD) in children aged 6–23 months is a simple qualitative indicator of the nutrient adequacy of the diet. An MDD indicator for children aged 6–23 months based on consumption of at least four out of seven food groups has been validated against the micronutrient density of child diet across several contexts (Moursi et al., 2008; Working Group on Infant and Young Child Feeding Indicators, 2006). The standard definition was recently updated to facilitate comparisons across breastfed and nonbreastfed children. Breast milk was added as an eighth food group and the threshold for MDD was raised to five out of eight food groups (World Health Organization & United Nations Children's Fund, 2017). Children 6–23 months old in sub‐Saharan Africa have some of the least diverse diets in the world (Choudhury et al., 2019). According to 2019 estimates published by UNICEF, one in four children 6–23 months old in Eastern and Southern Africa (ESA) attains MDD, which is only slightly better than one in five in South Asia (UNICEF, 2019). In the UNICEF Eastern and Southern Africa region, there has been a renewed effort to strengthen the role that regional economic communities or bodies such as the Southern African Development Community (SADC) and the Intergovernmental Authority on Development (IGAD) play to support country‐level efforts related to nutrition and food systems (Southern African Development Community, 2019).

Understanding the contextual factors that influence dietary diversity is important for informing action and programme design. As previously wealth and place of residence have been found to consistently correlate with diet diversity through 80 DHS analyses, and disparities in wealth and place of residence do exist in ESA, it becomes imperative to sift already available information considering these socioeconomic inequalities (Gatica‐Domínguez et al., 2021). Socioeconomic variables such as wealth, maternal education and exposure to media were associated with MDD and minimum acceptable diet among 77,887 weighted samples in sub‐Saharan countries (Belay, Aragaw, et al., 2022; Belay, Taddese, et al., 2022). Similarly, as predicted by Bennett's law, within countries, wealth is often positively correlated with dietary diversity, including for children (Bennett, 1941; Na et al., 2018; Nkoka et al., 2018). Differences also exist for urban versus rural residence: in a global analysis, the proportion of children from urban households reaching MDD was nearly twice that of rural children (39% vs. 23%) (UNICEF, 2021).

For selected countries in the ESA region, country‐specific analyses have identified risk factors or determinants associated with low diversity (Belew et al., 2017; Custodio et al., 2019; Nkoka et al., 2018), but variability in methods limit the cross‐country comparability of findings. There has been no systematic effort to date to identify common patterns in the risk factors of poor dietary diversity for children 6–23 months across ESA countries. Such an endeavour can be useful to guide regional efforts to share country learning and support countries with common challenges. This paper was prepared from a collaboration between United Nations International Children's Emergency Fund (UNICEF), John Hopkins University (JHU) and Global Alliance for Improved Nutrition (GAIN) focussed on the East and Southern Africa region, and was designed to develop the food policy guidance in those regions as a part of an effort to support cross‐country policymaking by regional governance bodies such as SADC and Intergovernmental Authority on Development (EGAD) (Ryckman et al., 2021; White et al., 2021).

This study has two related aims; (1) to use an equity lens to examine how MDD and consumption of specific food groups vary by wealth quintiles and urban/rural residence across nine countries in the ESA region and (2) to explore the common child, maternal and household‐level risk factors of not meeting MDD across countries.

## 2 METHODS

Nine countries out of 22 countries in the ESA region were selected based on the availability of DHS data, diversity in economic and food system characteristics, political actor's interest and link with another set of research activities being conducted by GAIN (Ryckman et al., 2021). For our analysis, UNICEF programming priorities and the desire to capture a variety of food system typologies were considered as it was part of an effort to input into regional governance bodies (SADC, EGAD). From Eastern Africa, Ethiopia (2016), Kenya (2014), Rwanda (2014–2015) and Uganda (2016) were, while from Southern Africa (2016), Malawi (2015–2016), Tanzania (2015–2016), Zambia (2015–2016) and Zimbabwe (2015) were included. These data sets were chosen based on the most recent one available in each country at the time the study was conducted. The DHS collects data on foods consumed in the past 24 h by the youngest child aged 6–23 months in each household. The selected countries and final sample size of children aged 6–23 months with MDD data are presented in Table 1.

**Table 1:** Household, parental and child characteristics among children 6–23 months of age in Eastern and Southern African countries.^a^

Characteristics	Ethiopia (*n* = 2965)	Kenya (*n* = 2906)	Malawi (*n* = 4879)	Rwanda (*n* = 2430)	South Africa (*n* = 872)	Tanzania (*n* = 3170)	Uganda (*n* = 4418)	Zambia (*n* = 3776)	Zimbabwe (*n* = 1656)
Area									
Rural	87.8	64.1	86.6	83.3	38.7	72.5	77.8	66.5	72.7
Urban	12.2	35.9	13.4	16.7	61.3	27.5	22.2	33.5	27.3
Female household head	13.5	29.0	24.7	20.4	52.8	17.2	23.5	18.4	37.3
Two of more children <5 years living in the same household	61.6	58.4	53.8	52.9	46.7	62.8	66.2	67.7	53.9
Improved drinking water source^b^	57.0	64.7	85.5	70.6	86.2	53.2	75.5	57.6	72.9
Improved toilet facility	10.4	46.3	81.6	30.9	69.9	26.8	33.0	38.7	56.5
Time to get to water source (min)									
Inside the house	12.1	28.6	12.4	8.8	73.2	22.0	14.9	19.5	30.6
1–59 min	58.5	53.5	63.4	69.2	19.0	51.9	55.9	67.9	51.4
≥60 min	29.4	19.9	24.2	22.0	7.8	26.1	29.2	12.6	18.0
Efficient cooking fuel^c^									
Efficient	3.4	15.4	1.2	0.3	78.4	1.2	0.4	7.7	23.3
Inefficient	96.6	84.7	98.8	99.7	21.6	98.8	99.6	92.3	76.7

^a^ All values (weighted %) are the ones accounted for survey design and sampling weights.

^b^ Improved drinking water sources piped into dwelling piped to yard/plot, public tap/standpipe, piped to the neighbour, tube well or borehole, protected well, protected spring, rainwater.

^c^ Efficient cooking fuel includes electricity, gas, and LPG while inefficient ones include charcoal, animal dung, wood, and others.

### 2.1 Outcome variables

The primary outcome of interest was the updated MDD indicator for children aged 6–23 months, reflecting the proportion of children reported to have consumed five or more of the following eight food groups in the previous 24 h: (1) grain (e.g., bread, rice, noodles, tubers), (2) legumes and nuts (made from beans, lentils, cowpeas), (3) vitamin A‐rich fruits and vegetables (e.g., pumpkin, orange‐fleshed sweet potatoes, green leafy vegetables), (4) other fruits and vegetables (e.g., bananas, avocados, tomatoes), (5) eggs, (6) flesh foods (beef, goat, chicken, organ meat and fish [Fresh or dried fish]), (7) dairy (milk, cheese, infant formula, yogurt) and (8) breast milk (UNICEF, 2019).

### 2.2 Analytical approach

**Figure 1:**
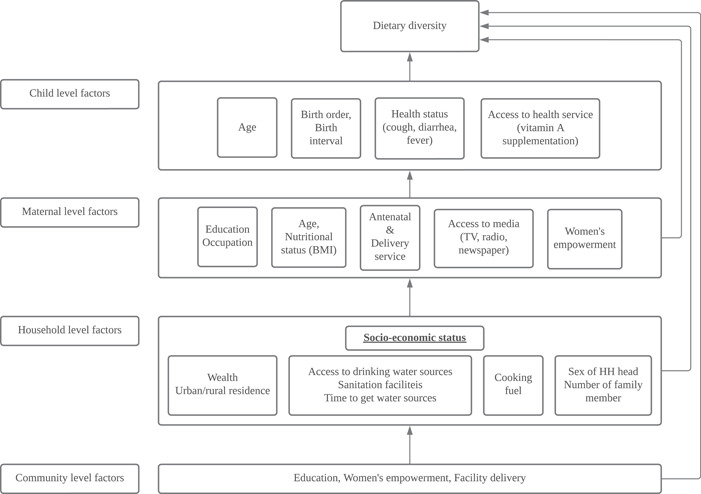
Conceptual framework of determinants of feeding practices among children above 6 months of age (modified from Blaney et al., 2015).

Based on a review of the literature (Blaney et al., 2015; Stewart et al., 2013) and inputs from the UNICEF Eastern and Southern Africa Regional Office, we identified a list of available DHS at the child‐ (*n* = 10), maternal‐ (*n* = 29), household‐ (*n* = 9) or community‐levels (*n* = 10) (Supporting Information: Table 1). The investigative team developed a conceptual framework (Figure 1) describing the relationships potentially influencing complementary feeding practices at each of these levels. We selected 35 candidate variables of greatest relevance to our study based on prior studies and our knowledge/UNICEF programming interest (Supporting Information: Table 2). The list of these variables at each level is presented below:

#### 2.2.1 Child characteristics

A total of six child‐level variables were identified: age (0–5, 6–23 or 24–59 month); birth order (1st, 2nd–4th or ≥5th), episodes of diarrhoea/cough/fever in the past 2 weeks, and vitamin‐A supplementation in the past 6 months.

#### 2.2.2 Maternal characteristics

The following 12 maternal‐level variables were identified: age (15–24, 25–34 or 35–49 years), highest education level, occupation (employment in agriculture, no formal occupation, waged occupation), body mass index (BMI) (<18.5, 18.5–25.0 or ≥25.0 kg/m^2^), number of antenatal visits during last pregnancy, places of recent delivery, delivery assisted by a health professional, watching TV, reading a newspaper, or listening to the radio at least once a week, exposure to any media (TV, newspaper or radio) at least once a week and women's empowerment. Maternal occupations included professional, clerical, sales, services and skilled and unskilled manual jobs. A score of women's empowerment (range: 0–5) was generated for each country using established methods, based on two domains of maternal participation in decision‐making and overall attitude towards domestic violence (Jennings et al., 2014). The score was then dichotomised into low (<50th percentile) and high (>50th percentile).

#### 2.2.3 Household characteristics

A total of eight household‐level variables were identified: residence (urban/rural), wealth quintile, sex of household head, number of children under 5 in the household, use of improved drinking water sources, use of improved sanitation facilities, time to access water sources and type of cooking fuel. Our analysis used the existing variables of county‐wide wealth quintiles generated by DHS itself through principal component analysis of household assets and infrastructure (Rutstein & Staveteig, 2014).

#### 2.2.4 Community characteristics

The following three community‐level variables were included; the proportion of women within the community who completed primary or higher education, the proportion of women within the community who gave birth at health facilities and the proportion of ‘empowered women’ within the community. The empowered women are those who belong to ‘high (>50th percentile)’ group, based on maternal participation in decision‐making and overall attitude towards domestic violence. The unit of community was the cluster (primary sampling unit) in the DHS data set. Each community variable was divided into tertiles (low, medium and high).

### 2.3 Statistical methods

For aim 1, the prevalence of MDD and consumption of specific food groups were estimated for the national sample and by wealth quintile (top vs. bottom) and by residence (rural vs. urban). The equity gap was calculated by taking the arithmetic difference in the prevalence of MDD and consumption of specific food groups between estimates for the upper and lowest wealth quintiles, and between rural and urban areas.

For aim 2, bivariate and multivariable logistic regression was conducted to estimate odds ratios and 95% confidence intervals (CI). Each of the candidate variables was included in bivariate logistic regression models as risk factors for not achieving MDD. Those that met a threshold for statistical significance of *p* < 0.05 were selected for inclusion in the final multivariable models for each country.

In some country models, decisions had to be made about retaining conceptually correlated variables. These included (1) for delivery place and receipt of assisted delivery by health professionals and (2) specific exposure to TV, newspaper or radio and to any of these media in the past week. For these sets of variables that were all significant in univariate analysis, the selection of which to include in the multivariable regression was based on at first, whether or not they remained significant in the multivariable model and then, which had the smaller *p* value when all sets of variables were significant in the multivariable model.

Only covariates with less than 10% missing value were included in the multivariable regression analysis. Variables that were statistically significant (*p* < 0.05) but with more than 10% missing in univariate regression were (1) women's empowerment in Kenya, South Africa and Zimbabwe, and (2) maternal BMI in Rwanda. Multicollinearity for the final multivariable logistic regression was checked by calculating variance inflation factors (VIF) and defined using a threshold of 4.0 for VIF and −0.2 for tolerance (Hair et al., 2010).

All analyses adjusted for DHS sampling design and survey weights were applied to generate nationally representative point estimates. All statistical analyses were conducted using Stata version 14 CI (StataCorp LP). Equiplots were used to visualise wealth and residence equity gaps (International Center for Equity in Health). Forest plots of bivariate associations between key risk factors (wealth quintiles, maternal age, child age and media exposure) and MDD were produced using GraphPad Prism 8.4.1 (https://www.graphpad.com/) A heatmap was generated using Microsoft Excel to show common risk factors with OR (95% confidence interval [CI]) among nine countries.

We defined ‘common regional risk factors of not meeting MDD’ across countries as those factors that were significant in the multivariable regression models of two or more countries.

### 2.4 Ethics and institutional review

The DHS data sets are publicly available from the DHS programme website (dhsprogram.com/data/). This secondary data analysis using deidentified data was deemed exempt from ethics review by the Institutional Review Board (IRB) at Johns Hopkins Bloomberg School of Public Health, USA.

## 3 RESULTS

### 3.1 Country demographics and socioeconomic status

Countries in the region exhibited similar patterns in their socioeconomic characteristics (Table 1). They were predominantly rural, less than 1/3 of households were female‐headed and more than half of households had at least two children under 5 years of age. Nearly all families used inefficient cooking methods while access to improved drinking water and sanitation was more variable. South Africa was the exception to the pattern with a high proportion of urban residents, a majority of female‐headed households and high access to improved drinking water in their houses and use of efficient cooking fuel. The mean child age was 14.0–14.4 months (range: 6–23)

### 3.2 Proportion of MDD and food groups

**Figure 2:**
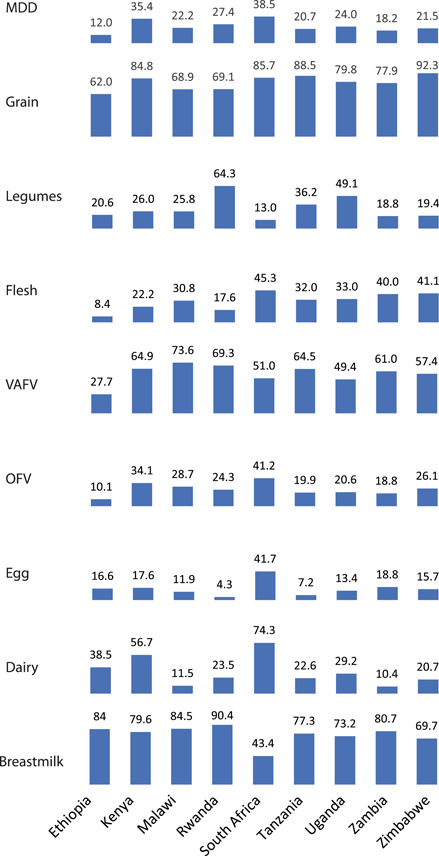
Minimum dietary diversity and food group consumption by children 6–23 months of age in nine Eastern and Southern African countries. Ethiopia 2016 (*n* = 2965); Kenya 2014 (*n* = 2906); Malawi 2015–2016 (*n* = 4879); Rwanda 2014–2015 (*n* = 2430); South Africa 2016 (*n* = 872); Tanzania 2015‐2016 (*n* = 3170); Uganda 2016 (*n* = 4418); Zambia 2013–2014 (3776); and Zimbabwe 2015 (*n* = 1656). The percentages represent the proportion of children consuming each item in the past 24 h reported by caregivers. All values were adjusted, and accounted for survey design and sampling weights. MDD, minimum dietary diversity; OFV, other fruits and vegetables; VAFV, vitamin A‐rich fruits and vegetables.

The proportion of children 6–23 months old attaining MDD was low across all nine countries. Only two countries exceeded 30%, South Africa (38.5%) and Kenya (35.4%) (Table 2 and Figure 2).

**Table 2:** Absolute difference (95% CI) in MDD between (A) richest and poorest wealth quintiles (B) rural and urban populations.

Country	MDD % pt	Grain % pt	Legume % pt	Flesh % pt	VAFV % pt	OFV % pt	Eggs % pt	Dairy % pt	Breast milk % pt
A. *Prevalence difference between top and bottom quintile* a, b									
Ethiopia	18.7 (14.8, 22.5)	20.0 (17.3. 22.0)	17.3 (15.1, 19.0)	11.4 (10.0, 12.4)	19.6 (18.5, 19.7)	14.0 (11.8, 15.4)	18.2 (16.1, 19.8)	12.8 (11.6, 13.1)	3.3 (2.6, 3.6)
Kenya	40.3 (35.7, 44.3)	14.0 (13.3, 13.8)	8.3 (4.8, 12.4)	13.6 (10.7, 16.6)	41.2 (40.0, 41.1)	39.3 (35.8, 42.1)	11.9 (8.5, 15.8)	22.2 (20.0, 23.5)	−3.7 (−7.6, −0.5)
Malawi	26.5 (23.8, 29.0)	14.8 (12.9, 16.0)	16.3 (14.5, 18.1)	21.5 (18.6, 24.3)	2.5 (1.6, 3.2)	12.0 (9.6, 14.4)	13.6 (11.1, 16.3)	29.5 (26.0, 32.9)	−7.3 (−9.7, −5.3)
Rwanda	30.5 (28.2, 32.5)	10.7 (10.1, 10.9)	−5.2 (−5.4, −4.9)	17.1 (15.8, 18.1)	10.9 (9.8, 11.6)	19.2 (16.1, 22.2)	10.5 (8.5, 12.6)	35.3 (31.8, 38.2)	−10.4 (−12.3, −8.6)
South Africa	14.4 (8.1, 19.9)	7.4 (4.4, 7.4)	−7.5 (−8.7, −2.7)	−3.2 (−7.9, 0.2)	24.1 (17.9, 27.3)	18.0 (11.7, 23.3)	13.2 (8.4, 17.1)	26.7 (22.5, 25.7)	−24.5 (−26.7, −19.8)
Tanzania	27.0 (24.2, 29.5)	7.8 (7.5, 7.5)	12.5 (11.9, 12.9)	32.7 (31.8, 32.8)	12.8 (12.0, 13.5)	17.6 (15.7, 19.3)	15.8 (12.1, 20.2)	8.0 (6.8, 9.3)	−5.3 (−5.8, −4.8)
Uganda	15.2 (13.1, 17.4)	7.2 (6.7, 7.4)	5.1 (2.7, 7.5)	9.5 (8.2, 10.7)	−15.2 (−16.0, −14.2)	11.9 (10.0, 13.9)	16.3 (14.5, 18.0)	37.9 (34.9, 40.5)	−17.6 (−18.6, −16.4)
Zambia	19.0 (16.2, 21.8)	5.9 (4.8, 6.5)	5.5 (3.7, 7.6)	23.9 (21.9, 25.6)	2.0 (0.6, 3.2)	9.8 (7.3, 12.7)	23.2 (19.3, 27.2)	28.3 (24.0, 32.5)	−27.6 (−30.6, −24.4)
Zimbabwe	31.1 (27.4, 34.0)	6.1 (5.3, 5.8)	11.6 (8.4, 15.1)	30.3 (27.8, 31.7)	−13.3 (−14.5, −11.8)	20.0 (17.8, 21.7)	22.7 (19.0, 26.3)	39.8 (36.2, 42.2)	−12.9 (−14.0, −11.5)
B. *Prevalence difference between urban and rural residence* a, b									
Ethiopia	17.5 (12.0, 24.1)	18.1 (15.5, 19.7)	12.6 (9.2, 16.3)	12.7 (10.4, 15.1)	18.2 (13.9, 22.5)	12.1 (8.1, 17.2)	11.8 (8.1, 16.1)	16.4 (11.5, 21.1)	−0.4 (−2.4, 2.4)
Kenya	22.7 (19.3, 26.0)	5.4 (4.8, 5.8)	4.6 (2.2, 7.4)	11.4 (9.2, 13.9)	21.5 (20.5, 22.0)	21.2 (18.3, 24.1)	6.1 (4.1, 8.6)	12.6 (10.9, 14.1)	−3.9 (−6.2, −2.0)
Malawi	18.1 (14.0, 22.4)	10.4 (6.2, 13.7)	11.8 (8.4, 15.6)	11.7 (7.8, 15.9)	2.3 (0.0, 4.2)	6.3 (2.1, 11.1)	7.9 (4.3, 12.5)	23.3 (18.7, 28.3)	−4.2 (−8.5, −0.8)
Rwanda	18.8 (16.1, 21.6)	9.7 (7.0, 11.7)	−5.2 (−7.2, −3.3)	11.4 (9.1, 13.9)	5.3 (2.9, 7.3)	11.7 (7.6, 16.1)	6.4 (4.6, 8.7)	17.9 (14.1, 21.9)	−6.6 (−9.0, −4.6)
South Africa	10.5 (10.2, 10.6)	0.2 (−0.1, 0.4)	−3.2 (−2.8, −3.5)	−1.9 (−2.3, −1.5)	13.7 (13.2, 13.8)	6.7 (5.8, 7.2)	7.8 (6.7, 8.5)	17.0 (15.8, 17.8)	2.7 (2.5, 2.8)
Tanzania	14.7 (12.5, 17.1)	4.4 (3.3, 5.0)	6.5 (5.1, 7.9)	17.7 (15.4, 20.0)	8.4 (7.0, 9.6)	8.9 (7.0, 11.0)	8.9 (6.3, 12.3)	3.3 (1.7, 5.2)	−5.6 (−7.0, −4.3)
Uganda	5.5 (3.4, 7.9)	5.3 (3.5, 6.6)	0.5 (−2.3, 3.4)	6.2 (4.6, 8.0)	−9.2 (−10.9, −7.4)	5.4 (2.9, 8.3)	7.8 (5.8, 10.1)	19.5 (17.0, 22.0)	−11.2 (−13.6, −8.8)
Zambia	9.0 (7.3, 10.9)	7.8 (7.5, 7.8)	2.8 (2.0, 3.8)	12.0 (10.7, 13.3)	−1.0 (−2.3, 0.3)	5.9 (4.4, 7.6)	11.2 (9.4, 13.3)	15.1 (12.9, 17.6)	−16.2 (−17.9, −14.5)
Zimbabwe	20.2 (17.1, 23.3)	2.2 (0.9, 2.8)	5.7 (3.5, 8.4)	24.4 (23.1, 25.4)	−8.9 (−11.3, −6.4)	16.5 (14.6, 18.3)	15.3 (13.1, 17.6)	29.9 (26.9, 32.6)	−11.8 (−13.8, −9.8)

Abbreviations: CI, confidence interval; MDD, minimum dietary diversity; OFV, other fruits and vegetables; pt, percentage point; VAFV, vitamin‐A fruits and vegetables.

^a^ All values (weighted % [95% CI]) were estimated accounted for survey design and sampling weights.

^b^ Positive numbers indicate that wealthier households have a greater prevalence of attaining MDD (A), and that urban households have a greater prevalence of attaining MDD (B), and negative numbers indicate the opposite.

The ‘grains’ food group was the most commonly consumed food group across all nine countries. The proportion of children consuming breast milk exceeded 70% in nearly all countries but was notably low in South Africa (43.4%). Legume consumption was highest in Rwanda (64.3%), and lowest in South Africa (13.0%). Pro‐vitamin A‐rich fruits and vegetables (VAFV) were commonly consumed in Malawi (73.6%) and Rwanda (69.3%); moderately in Uganda (49.4%) and Zimbabwe (57.4%); and lowest in Ethiopia (27.7%). Other fruits and vegetables (OFV) were less likely to be consumed than VAFV in all nine countries; children in Ethiopia had the lowest reported OFV consumption (10.1%). Flesh foods (meat, fish and organ meat) were more commonly consumed by children in countries within the Southern African region (ranging from 45.3% in South Africa to 25.8% in Malawi) than the Eastern African region (ranging from 33.3% in Uganda to 8.4% in Ethiopia). Egg consumption was less than 20% in all countries except South Africa (41.7%). Dairy food consumption varied across countries: Zambia (10.4%) had the lowest consumption and South Africa had exceptionally high consumption (74.3%).

**Figure 3:**
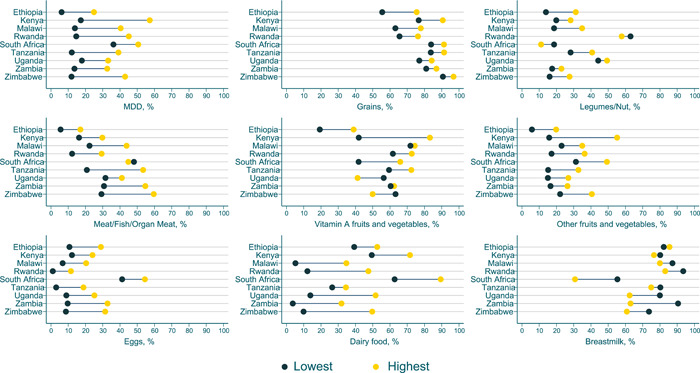
Equiplots of eight food groups and minimum dietary diversity between richest and poorest household wealth quintiles in nine Eastern and Southern African countries. All values were estimated accounted for survey design and sampling weights.

### 3.3 Equity gaps in MDD and food group consumption

**Figure 4:**
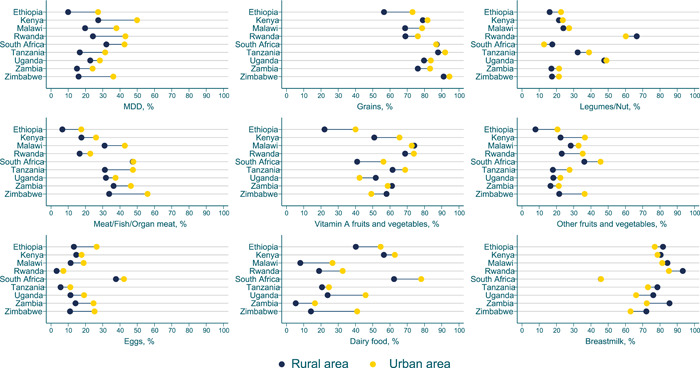
Equiplots of eight food groups and minimum dietary diversity by urban/rural residence in nine Eastern and Southern African countries. All values were estimated accounted for survey design and sampling weights.

In all nine countries, a greater proportion of children met the threshold for MDD in wealthier households compared with poorer households (Figures 3 and 4, Table 2 and Supporting Information: Table 3). The equity gap for MDD by wealth status was largest in Kenya (40 pp), Zimbabwe (31.1 pp) and Rwanda (30.5 pp); smaller gaps were observed in Uganda (15.2 pp), South Africa (14.4 pp) and Zambia (19.0 pp) (all *p* < 0.05 but South Africa). A similar pattern was observed for individual food groups, with the exception of breast milk, which in all countries except Ethiopia was reported more frequently by poorer households (Figure 4). In both Zimbabwe and Uganda (−13.3 and −15.2 pp, respectively; all *p* < 0.05), poorer households were more likely to consume VAFV than wealthier households, but the gap was minor in Zambia or Malawi. For both the flesh foods category (meat/fish/or organ meat; −3.2, −32.7 pp) and the dairy food category (8.0, −39.9 pp), equity gaps were particularly large in all countries (*p* < 0.05), with the exception of South Africa (*p* > 0.05), which had slightly higher consumption of flesh foods by poor households than wealthy households. In Rwanda, a greater proportion of children from poorer households also consumed legumes than those from wealthier households (−5.2 pp; *p* < 0.05).

Additional equity dimensions in consumption were apparent in equiplots of MDD and food group consumption by urban/rural residences (Figure 2 and Supporting Information: Tables 2 and 3). Children from urban households were more likely to attain MDD than those from rural households and a similar pattern held for most foods in all nine countries (5.5, −22.7 pp; all *p* < 0.05). Generally, the urban–rural gap for MDD‐specific food groups was not as large as the gap between the lowest and highest wealth quintile as the standard of living of the urban population is better than that of the rural areas. It is notable that vegetable consumption was less equitable across rural and urban categories, compared with other food groups, particularly in Malawi and Zambia. South Africa had the second‐largest equity gap in breast milk consumption by wealth (−24.5 pp; *p* < 0.05) but no meaningful gap was apparent between urban and rural areas. Patterns of equity by wealth using the current MDD indicator were similar to those observed using the prior seven group indicator that did not consider breastfeeding (Supporting Information: Table 4).

### 3.4 Risk factor analysis

**Figure 5:**
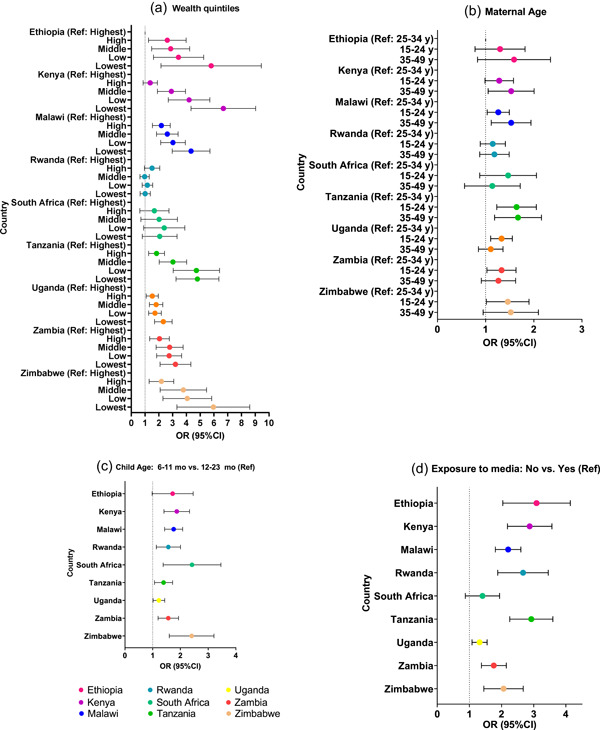
Forest plots of bivariate analysis of the relationship between key factors and odds of not meeting the MDD threshold. ^1^Odds ratios >1.0 indicate less likely to attain MDD compared with a reference group. All values were estimated accounted for survey design and sampling weights. MDD, minimum dietary diversity; OR, odds ratio.

In bivariate models, the direction of the association between MDD and wealth quintiles (except for Rwanda), maternal age, media and child age was consistent in all nine countries (Figure 5). Younger children (6–11 vs. ref: 12–23 months; Figure 5a), those from lower wealth quintiles (Figure 5b), those with youngest (15–24 years) or oldest (35–49 years) mothers (ref: 25–34 years; Figure 5c) and those with no recent maternal exposure to any media (Figure 5d) were more likely to not achieve MDD.

**Figure 6:**
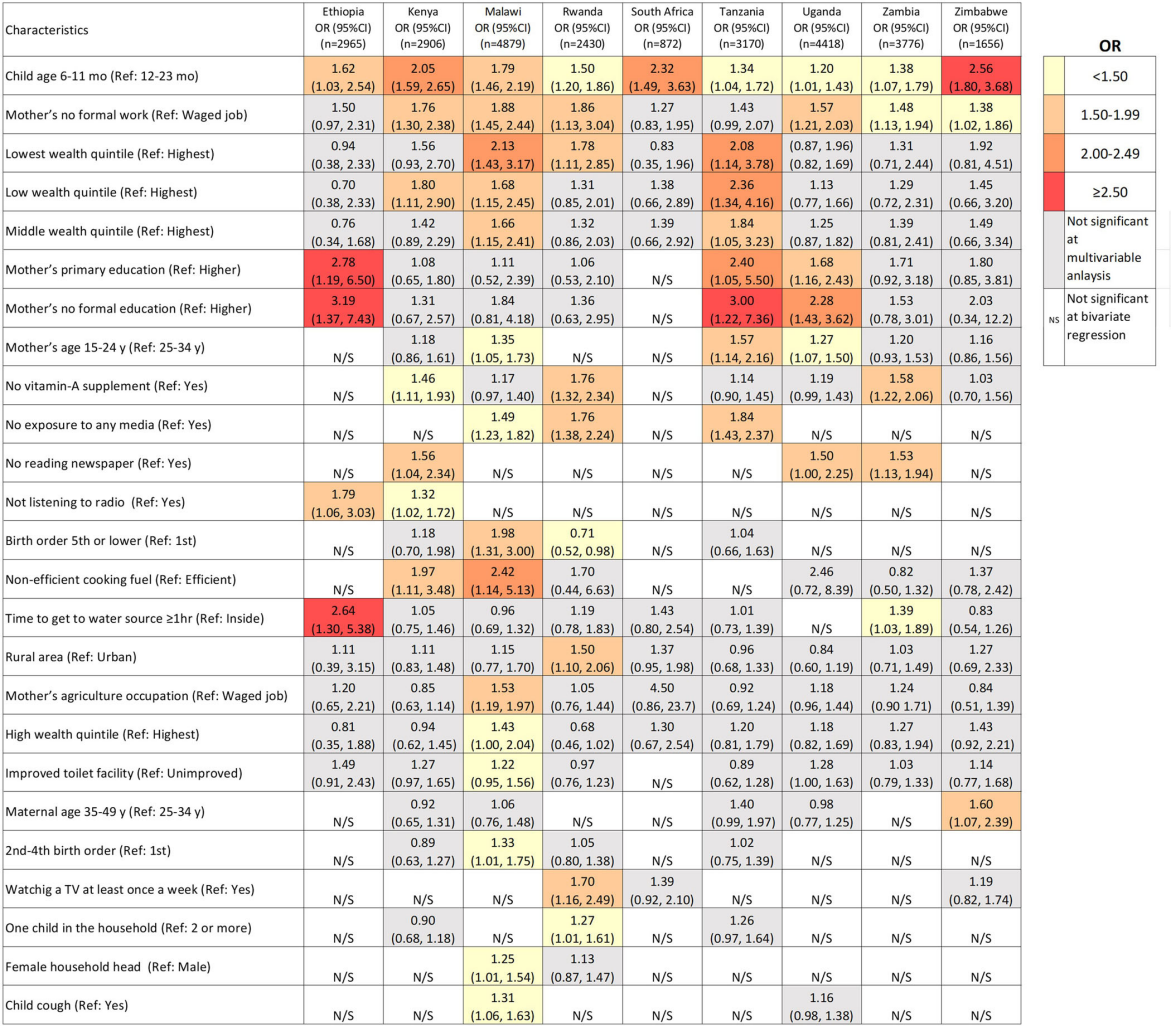
Multivariable models of the odds of not reaching minimum dietary diversity^1^ in nine Eastern and Southern African countries. Odds ratios >1.0 indicate less likely to attain MDD compared with the reference group. Table only presents variables that were significant in ≥2 country‐specific multivariable regression models; n/a indicates that the variable was not significant in bivariate analysis. All values were estimated accounted for survey design and sampling weights. The threshold used for each colour was decided arbitrary through discussion by the authors. MDD, minimum dietary diversity; N/S; not significant at bivariate regression; OR, odds ratio.

In multivariable models, a number of common risk factors for not meeting MDD were identified across two or more countries (Figure 6 and Supporting Information: Table 5). In all nine countries, younger child age (6–11 months) was associated with greater odds of not meeting MDD compared with ages 12–23 months (OR range: 1.20–2.57). Relative to mothers with a waged job, those without formal work were less likely to achieve MDD in six countries (OR range: 1.37‐1.88). In three countries, Tanzania, Malawi and Rwanda, children from households in the lowest wealth quintile had from 1.8 to 2.1 times higher odds of not meeting MDD compared to the highest quintile.

Compared with higher education, informal maternal education or primary education was associated with increased odds of not achieving MDD (OR range: 2.28–3.19 times and 1.68–2.78 times) in Ethiopia, Tanzania and Uganda. No exposure to media (radio, tv or newspaper) in the past week was associated with higher odds of not meeting MDD in Malawi, Rwanda and Tanzania odds ratio (OR range: 1.49–1.80). Not reading a newspaper in the past week was associated with not meeting MDD in Zambia and Kenya.

Maternal young age (15–24 years), compared with maternal age of 25–34 years, was associated with higher odds of not meeting MDD in Tanzania, Malawi and Uganda. Not having received vitamin‐A supplementation among children in the past 6 months was associated with not achieving MDD in Rwanda, Zambia and Kenya. The use of inefficient cooking fuel was associated with higher odds of not meeting MDD in Malawi and Kenya. Compared with using water inside the households, households spending greater than 1 h to collect drinking water had a higher risk of not attaining MDD in Ethiopia and Zambia. Compared with first‐born children, the birth order ≥ 5 imposed a higher risk of not attaining MDD by 1.98 times (95% CI: 1.31, 3.00) in Malawi but a lower risk in Rwanda (OR: 0.71, 95% CI: 0.52, 0.98).

## 4 DISCUSSION

This study systematically examined inequities and risk factors related to child dietary diversity, across multiple countries in the ESA region. The findings reveal the presence of important inequities in dietary diversity across the ESA region by both wealth status and rural/urban location. A number of factors were identified as predictors of MDD across multiple countries including the mother's employment, household wealth, mother's education, exposure to different types of media and/or access to water sources and efficient cooking fuel.

Our findings were similar to those from a study from 80 low‐and‐middle‐income countries which observed wealth inequities in child consumption of certain food groups including dairy, flesh foods, eggs and to other fruits and vegetables but no differences for cereals, legumes and VAFV (Gatica‐Domínguez et al., 2021).

Consumption of animal‐source foods (ASF) was generally low in most households across the region except for South Africa and egg consumption was less frequent than other types of animal‐source foods. There has been a recent focus on improving egg consumption to improve child nutrition in low‐ and middle‐income countries (C. K. Lutter et al., 2018). Data suggest that the supply of eggs is relatively scarce in Africa, where only 2.5 kg of eggs per person were available compared with the global average of 8.9 and 9.1 kg in Asia (FAO, 2010). Given the challenges of keeping chickens healthy in‐home production systems, investments in commercial production may be needed to lower the price of eggs and increase accessibility (C. Lutter et al., 2020; C. K. Lutter, 2020).

Fish are an important but underutilized resource for addressing nutrient gaps, particularly in coastal countries in the region (FAO, 2020). Poultry is relatively more affordable in Southern Africa than in Eastern Africa which may explain the differences we observed in reported consumption of flesh foods (OECD/FAO., 2016). Ownership of cows or livestock is associated with higher consumption of dairy and meat foods among household members but may be less accessible to young children (Haileselassie et al., 2020; Hetherington et al., 2017). Given that different animal source foods can provide different under‐consumed nutrients, the ESA region should prioritise identifying strategies to increase consumption of multiple ASFs as fit for the local context rather than focussing on a single type of ASF. Additionally, given that the links between farm production and dietary diversity are positive but typically small (Ruel et al., 2018; Sibhatu & Qaim, 2018), market‐based interventions possibly including conditional cash transfers may be needed to enhance access to diverse diets (Manley et al., 2022).

Interesting patterns were also apparent for provitamin‐A‐rich fruit and vegetable consumption. Malawi and Rwanda both had relatively high and equitable consumption relative to other countries. Kenya and Tanzania also had over 60% consumption, but consumption patterns were not as equitable by wealth or urban/rural residence. It would be useful to better understand the household production, accessibility and affordability of these fruits and vegetables across contexts and to identify whether there are specific strategies in Malawi and Rwanda that can be replicated. Unlike other countries, in Zimbabwe and Uganda, children from poorer households and rural households consumed more VAFV than wealthier or urban households. Uganda ranks as the second largest producer of fruits and vegetables in sub‐Saharan Africa, with horticulture in all districts (Dijkxhoorn et al., 2019). It may be that a more robust fruit farming sector could help explain the different trends in these countries (Dijkxhoorn et al., 2019; Zimbabwe National Statistics Agency, 2019). A study examining the cost of purchasing nutritious complementary foods in six ESA countries reported that vitamin A is an affordable nutrient through purchases of dark leafy greens, orange‐fleshed vegetables and liver (Ryckman et al., 2021).

Breast milk was consistently more commonly consumed by children from both poorer and rural households. Conversely, the timely introduction of complementary feeding seemed to be higher in urban and wealthier households (Supporting Information: Table 6). This trend indicates breastfeeding or delay in complementary foods are driven by accessibility and affordability of foods for the child. In Kenya, continued breastfeeding, timely introduction of complementary foods and dietary diversity are more balanced than other countries (Supporting Information: Table 6 and Figure 2). This is likely driven in part by employment patterns: other studies have shown that having an occupation in the public or private sector is associated with early cessation of exclusive breastfeeding and earlier introduction of complementary foods (Coulibaly et al., 2014; Habtewold et al., 2019). At a regional level, efforts to support countries by strengthening maternity protection laws or even creating family–friendly workspaces that enable women to continue to breastfeed their children can be a way of supporting breastfeeding for working women in urban areas (UNICEF, 2019).

The Republic of South Africa stood out as having the highest MDD rate, the smallest MDD equity gap and a much higher rate of urbanisation compared with the other eight countries in our analysis. Given the comparatively advanced state of economic development in South Africa compared with the rest of the region, the patterns seen in South Africa could provide an indication of where diets in other countries in the region are heading. South Africa had the highest reported consumption of all three animal‐source food groups, notably eggs and dairy. However, South Africa also had the lowest reported consumption of breast milk and legumes, particularly among the wealthiest and urban households. Given the lower cost and the dual benefits related to health and sustainability associated with the consumption of these two items, it may be worthwhile trying to promote greater consumption of legumes and breast milk among young children. In South Africa, unlike other countries, maternal education or traditional media exposure was not associated with dietary diversity. This may have to do with the better economy, infrastructure, and access to various social media in South Africa where mothers can be knowledgeable and afford to feed their kids a variety of foods than other ESA countries.

Ethiopia had the lowest rate of MDD and lowest rates of consumption for several food groups including grains, meat and fish and any fruits and vegetables. The Ethiopian Orthodox church, which is 43.5% of the population (Ethiopian Central Statistical Agency, 2012), requires more than 200 days of fasting per year from most animal‐source foods, which includes all meat and fish. Young children are supposed to be excluded from this requirement, but their diets may be subject to wider household practices. Religious leaders have been considered a key target of IYCF programming for this reason (Sanghvi et al., 2013). Ethiopia is also the farthest north of the ESAR countries and, similar to Kenya, includes large pastoralist regions. Livestock ownership is high but studies suggest that they are not slaughtered for household consumption (Bundala et al., 2020); however, they may be a good food source to provide milk or eggs to children. Promoting the consumption of ASF in Ethiopia would be a reasonable choice to provide animal protein and micronutrients such as iron.

Rwanda ranks as having among the highest consumption of beans in the world (~29 kg per capital per year), and about 20% of the beans produced are iron‐rich varieties (Larochelle & Alwang, 2014). Given the low consumption of this food group across other countries, learning from Rwanda about how to enhance the production and consumption of beans and legumes is an important opportunity for other countries in the region.

Consistent with global findings, younger children (6–11 months) had a less diverse diet compared with older ages (12–24 months). During this early period, children often lack teeth to chew and there may be concerns about feeding young children foods that require chewing (Mura Paroche et al., 2017). Younger children would be at greater risk of MDD failure if the timely initiation of complementary feeding is delayed (Supporting Information: Table 6). As premastication has become less common, foods such as meat or fish require extra preparation, which can be taxing in contexts where mothers lack labour‐saving technologies (Birch et al., 2007; Pelto et al., 2010). Also, until quite recently, counselling and communication messages about complementary feeding have emphasised the gradual stepwise introduction of foods to ensure children tolerate and are not allergic to foods (Mura Paroche et al., 2017). Thus, counselling and communication messages may need to be adjusted to emphasise the importance of dietary diversity among young children, along with efforts to increase availability and access to animal‐source foods and to address preparation challenges. While not necessarily increasing overall diversity scores, use of fortified cereals is another strategy to iron and zinc deficiencies in younger children in settings where access to animal‐source foods or preparation methods are challenging (Allen et al., 2006).

The finding that receipt of vitamin‐A supplementation was associated with higher MDD is likely indicative of better access to health services where SBCC or IYCF counselling may be provided. In many countries, higher media access was associated with higher MDD, a finding that could have multiple explanations including access to information about the importance of dietary diversity for young children. This finding also supports the need to explore the use of multiple communication channels that are context‐specific and reach the maximum number of mothers, caregivers and other influencers such as fathers and grandmothers.

The type of media related to MDD varies by country. Radio may be an important source of information in Ethiopia and Malawi, but newspapers may be a dominant factor in Kenya, Uganda, and Zambia. Wealthier households are more likely to have frequent exposure to various media sources. With an increase in mobile phone use and internet access, there is an opportunity to potentially improve dietary quality in resource‐poor settings (Flax et al., 2022). At the same time, mass media can increase exposure to messaging related to commercial milk formula and other commercial complementary foods with low nutrient density in violation of WHO breastfeeding and complementary feeding guidelines (Cetthakrikul et al., 2022). Thus, it is important that strategies to use media to promote proper infant and young child feeding practices encompass the examination of a broad set of opportunities that are aligned with WHO guidelines.

Low incomes and heavy workload for women are linked with poor child feeding across the region (Ahishakiye et al., 2019; Chakona, 2020; Solomon et al., 2017). Findings from this study highlight that an investment in maternal education, keeping girls in school and ensuring completion of secondary education should be prioritised in the region as part of the collaboration with the education sector. In three countries (Tanzania, Malawi, Uganda), we found that children of the youngest maternal age group (15–24 years old) had significantly higher odds of not meeting MDD than children of older women. ESA has one of the highest rates of adolescent pregnancy and young motherhood in the world (Africa, 2020) and young mothers are less likely to use timely antenatal and postnatal care and have limited access to nutrition information (Mekonnen et al., 2019). Approaches that reach adolescent mothers using existing community systems and structures and social media channels could be an entry point for ensuring these vulnerable girls are reached with IYCF counselling and support. Additionally, given that elders or grandparents may play a particularly significant role in the lives of adolescent mothers and their infants, it may be worth developing approaches to reach them with counselling and support messages as well (Bray & Dawes, 2016).

Despite sharing many determinants of MDD with varying degrees of association, we found that some determinants varied across countries. In South Africa, unlike other countries, maternal education or traditional media exposure was not associated with dietary diversity. This may have to do with the better economy, infrastructure and access to various social media in South Africa where mothers can be knowledgeable and afford to feed their kids a variety of foods than other ESA countries. The type of media related to MDD varies by country. Radio may be an important source of information in Ethiopia and Malawi, but newspapers may be a dominant factor in Kenya, Uganda and Zambia.

Key strengths of this study included the use of nationally representative data spanning nine countries in the ESA region and the use of the revised 8‐item MDD score. However, the generalisability of the findings to the remaining countries in the region is uncertain. Due to the limited information in the DHS related to food systems (including access to food), we were unable to examine a number of risk factors for MDD that we would have anticipated being possible drivers of low MDD, including proximity to markets, market prices and availability of foods in the market. The cross‐sectional nature of the analysis also limits the ability to draw causal inferences between risk factors and MDD. Since MDD is compared across countries at different timepoints, we may not exclude potential biases related to seasonal or yearly differences in food availability. For example, an assessment made at harvest seasons results in narrower wealth gaps in some crops. While we strived to use the most available DHS data for the countries at the time of the analysis, in three countries, a more recent data set is now available, and the generalisability of our findings to that survey is uncertain.

## 5 CONCLUSIONS

Meaningful differences in the rate of MDD and wide equity gaps were apparent for most food groups between high and low‐wealth quintiles and rural and urban populations in ESA. We identified many common socioeconomic factors associated with child dietary diversity across countries. Such common factors across countries provide grounding for regional actions to address infant and young child feeding. Interventions to address when complementary foods are introduced, support adolescent mothers or mothers with poor education, and improve food security may be prioritised for child nutrition programmes.

## AUTHOR CONTRIBUTIONS

Yunhee Kang was involved in conceptualization, methodology, formal analysis, writing of the manuscript–original draft, and investigation. Rebecca A. Heidkamp was involved in conceptualisation, writing of the manuscript–review and editing, and investigation. Kudakwashe Mako‐Mushaninga was involved in conceptualization and writing of the manuscript–review and editing. Aashima Garg was involved in conceptualization and writing–review and editing. Joan N. Matji was involved in conceptualization and writing–review and editing. Mara Nyawo was involved in writing of the manuscript–review and editing. Hope C. Craig was involved in writing of the manuscript–review and editing. Andrew L. Thorne‐Lyman was involved in conceptualisation, methodology, writing of the manuscript–original draft, funding acquisition, and project administration.

## CONFLICT OF INTEREST STATEMENT

The authors declare no conflicts of interest.

## Supporting information

Supporting information.

## Data Availability

Data that support the findings of this study are openly available at https://dhsprogram.com/data

## References

[mcn13487-bib-0002] Ahishakiye, J. , Bouwman, L. , Brouwer, I. D. , Matsiko, E. , Armar‐Klemesu, M. , & Koelen, M. (2019). Challenges and responses to infant and young child feeding in rural Rwanda: A qualitative study. Journal of Health, Population, and Nutrition, 38(1), 43. 10.1186/s41043-019-0207-z 31831068 PMC6907215

[mcn13487-bib-0003] Allen, L. , de Benoist, B. , Dary, O. , & Hurrell, R. (2006). Guidelines on food fortification with micronutrients. World Health Organization/Food and Agriculture Organization.

[mcn13487-bib-0004] Belay, D. G. , Aragaw, F. M. , Teklu, R. E. , Fetene, S. M. , Negash, W. D. , Asmamaw, D. B. , Fentie, E. A. , Alemu, T. G. , Eshetu, H. B. , & Shewarega, E. S. (2022). Determinants of inadequate minimum dietary diversity intake among children aged 6‐23 months in Sub–Saharan Africa: Pooled prevalence and multilevel analysis of demographic and health survey in 33 Sub–Saharan African countries. Frontiers in Nutrition, 9, 894552. 10.3389/fnut.2022.894552 35845763 PMC9284213

[mcn13487-bib-0005] Belay, D. G. , Taddese, A. A. , & Gelaye, K. A. (2022). Minimum acceptable diet intake and its associated factors among children age at 6‐23 months in sub–Saharan Africa: A multilevel analysis of the sub–Saharan Africa demographic and health survey. BMC Public Health, 22(1), 684. 10.1186/s12889-022-12966-8 35392871 PMC8991979

[mcn13487-bib-0006] Belew, A. K. , Ali, B. M. , Abebe, Z. , & Dachew, B. A. (2017). Dietary diversity and meal frequency among infant and young children: A community based study. Italian Journal of Pediatrics, 43(1), 73. 10.1186/s13052-017-0384-6 28810887 PMC5558775

[mcn13487-bib-0007] Beluska‐Turkan, K. , Korczak, R. , Hartell, B. , Moskal, K. , Maukonen, J. , Alexander, D. E. , Salem, N. , Harkness, L. , Ayad, W. , Szaro, J. , Zhang, K. , & Siriwardhana, N. (2019). Nutritional gaps and supplementation in the first 1000 days. Nutrients, 11(12), 2891. 10.3390/nu11122891 31783636 PMC6949907

[mcn13487-bib-0008] Bennett, M. K. (1941). International contrasts in food consumption. Geographical Review, 31(3), 365–376.

[mcn13487-bib-0009] Birch, L. , Savage, J. S. , & Ventura, A. (2007). Influences on the development of Children's eating behaviours: From infancy to adolescence. Canadian Journal of Dietetic Practice and Research: A Publication of Dietitians of Canada = Revue Canadienne de la Pratique et de la Recherche en Dietetique: Ane Publication des Dietetistes du Canada, 68(1), 1. PMC2678872 19430591

[mcn13487-bib-0010] Blaney, S. , Februhartanty, J. , & Sukotjo, S. (2015). Feeding practices among Indonesian children above six months of age: A literature review on their magnitude and quality (part 1). Asia Pacific Journal of Clinical Nutrition, 24(1), 16–27. 10.6133/apjcn.2015.24.1.13 25740738

[mcn13487-bib-0103] Bray, R. , & Dawes, A. (2016). *Parenting, Family Care and Adolescence in East and Southern Africa: An evidence‐focused literature review* (Innocenti Discussion Papers, no. 2016‐02). UNICEF Office of Research‐Innocenti.

[mcn13487-bib-0011] Bundala, N. , Kinabo, J. , Jumbe, T. , Rybak, C. , & Sieber, S. (2020). Does homestead livestock production and ownership contribute to consumption of animal source foods? A pre‐intervention assessment of rural farming communities in Tanzania. Scientific African, 7, e00252.

[mcn13487-bib-0013] Cetthakrikul, N. , Kelly, M. , Baker, P. , Banwell, C. , & Smith, J. (2022). Effect of baby food marketing exposure on infant and young child feeding regimes in Bangkok, Thailand. International Breastfeeding Journal, 17(1), 64. 10.1186/s13006-022-00503-7 36050746 PMC9435428

[mcn13487-bib-0014] Chakona, G. (2020). Social circumstances and cultural beliefs influence maternal nutrition, breastfeeding and child feeding practices in South Africa. Nutrition Journal, 19(1), 47. 10.1186/s12937-020-00566-4 32434557 PMC7240933

[mcn13487-bib-0016] Choudhury, S. , Headey, D. D. , & Masters, W. A. (2019). First foods: Diet quality among infants aged 6‐23 months in 42 countries. Food Policy, 88, 101762. 10.1016/j.foodpol.2019.101762 31853163 PMC6894322

[mcn13487-bib-0018] Coulibaly, A. , Ake Tano, O. , Benie Bi Vroh, J. , Traore, Y. , & Dagnan, N. S. (2014). [Socioeconomic factors influencing exclusive breastfeeding among primiparous women in Abidjan (Ivory Coast)]. Sante Publique, 26(4), 555–562. 25380271

[mcn13487-bib-0019] Custodio, E. , Herrador, Z. , Nkunzimana, T. , Węziak‐Białowolska, D. , Perez‐Hoyos, A. , & Kayitakire, F. (2019). Children's dietary diversity and related factors in Rwanda and Burundi: A multilevel analysis using 2010 Demographic and Health Surveys. PLoS One, 14(10), e0223237. 10.1371/journal.pone.0223237 31596868 PMC6785172

[mcn13487-bib-0020] Dijkxhoorn, Y. V. G. M. , Barungi, J. , Okiira, J. , Gema, J. , & Janssen, V. (2019). *The Uganda vegetables and fruit sector: Competitiveness, investment and trade options*. Wageningen, Wageningen Economic Research, Report 2019‐117.

[mcn13487-bib-0021] Ethiopian Central Statistical Agency . (2012). *2007 Population and Housing Census of Ethiopia*. Addis Ababa.

[mcn13487-bib-0022] FAO . (2010). Poultry Meat & Eggs: Agribusiness Handbooks.

[mcn13487-bib-0023] FAO . (2020). The State of World Fisheries and Aquaculture 2020. Sustainability in action.

[mcn13487-bib-0024] Flax, V. L. , Ipadeola, A. , Schnefke, C. H. , Kwasu, S. , Mikail, A. A. , Bose, S. , Brower, A. O. , & Edwards, S. (2022). Complementary feeding social and behavior change communication for fathers and mothers improves children's consumption of fish and eggs and minimum meal frequency in Kaduna State, Nigeria. Current Developments in Nutrition, 6(5), 6005012. 10.1093/cdn/nzac075 PMC9154220 35669047

[mcn13487-bib-0026] Gatica‐Domínguez, G. , Neves, P. A. R. , Barros, A. J. D. , & Victora, C. G. (2021). Complementary feeding practices in 80 low‐ and Middle‐income countries: Prevalence of and socioeconomic inequalities in dietary diversity, meal frequency, and dietary adequacy. The Journal of Nutrition, 151(7), 1956–1964. 10.1093/jn/nxab088 33847352 PMC8245881

[mcn13487-bib-0027] Habtewold, T. D. , Mohammed, S. H. , Endalamaw, A. , Akibu, M. , Sharew, N. T. , Alemu, Y. M. , Beyene, M. G. , Sisay, T. A. , Birhanu, M. M. , Islam, M. A. , & Tegegne, B. S. (2019). Breast and complementary feeding in Ethiopia: New national evidence from systematic review and meta‐analyses of studies in the past 10 years. European Journal of Nutrition, 58(7), 2565–2595. 10.1007/s00394-018-1817-8 30229308

[mcn13487-bib-0029] Haileselassie, M. , Redae, G. , Berhe, G. , Henry, C. J. , Nickerson, M. T. , Tyler, B. , & Mulugeta, A. (2020). Why are animal source foods rarely consumed by 6‐23 months old children in rural communities of Northern Ethiopia? A qualitative study. PLoS One, 15(1), e0225707. 10.1371/journal.pone.0225707 31914130 PMC6948827

[mcn13487-bib-0030] Hair, J. B. W. C. , Babin, B. J. , & Anderson, R. E. (2010). Multivariate data analysis (7th ed.). Pearson Education International.

[mcn13487-bib-0031] Hetherington, J. B. , Wiethoelter, A. K. , Negin, J. , & Mor, S. (2017). Livestock ownership, animal source foods and child nutritional outcomes in seven rural village clusters in sub‐Saharan Africa. Agriculture & Food Security, 6, 9.

[mcn13487-bib-0033] Jennings, L. , Na, M. , Cherewick, M. , Hindin, M. , Mullany, B. , & Ahmed, S. (2014). Women's empowerment and male involvement in antenatal care: Analyses of demographic and Health Surveys (DHS) in selected African countries. BMC Pregnancy and Childbirth, 14, 297. 10.1186/1471-2393-14-297 25174359 PMC4161883

[mcn13487-bib-0035] Kathryn G., D. (2013). The challenge of meeting nutrient needs of infants and young children during the period of complementary feeding: An evolutionary perspective. The Journal of Nutrition, 143(12), 2050–2054. 10.3945/jn.113.182527 24132575 PMC3827643

[mcn13487-bib-0038] Kuklina, E. V. , Ramakrishnan, U. , Stein, A. D. , Barnhart, H. H. , & Martorell, R. (2004). Growth and diet quality are associated with the attainment of walking in rural Guatemalan infants. The Journal of Nutrition, 134(12), 3296–3300. 10.1093/jn/134.12.3296 15570028

[mcn13487-bib-0039] Larochelle, C. , & Alwang, J. (2014). Impact of adopting improved bean varieties on poverty and food security in Rwanda. ISPC Secretariat, Rome. Paper presented at the Agricultural & Applied Economics Association's 2014 AAEA Annual Meeting, Minneapolis, MN.

[mcn13487-bib-0040] Lutter, C. , Caswell, B. , Arnold, C. , Iannotti, L. , Prado, E. , Maleta, K. , Chipatala, R. , & Stewart, C. (2020). The effect of providing eggs early in complementary feeding on energy intake and dietary diversity: The Mazira project randomized controlled trial. Current Developments in Nutrition, 4(Suppl 2), 863.

[mcn13487-bib-0041] Lutter, C. K. (2020). Building the evidence base around poultry production for nutrition. The Journal of Nutrition, 150, 2617–2618. 10.1093/jn/nxaa247 32856076

[mcn13487-bib-0042] Lutter, C. K. , Iannotti, L. L. , & Stewart, C. P. (2018). The potential of a simple egg to improve maternal and child nutrition. Maternal & Child Nutrition, 14(Suppl 3), e12678. 10.1111/mcn.12678 30332538 PMC6865885

[mcn13487-bib-0043] Manley, J. , Alderman, H. , & Gentilini, U. (2022). More evidence on cash transfers and child nutritional outcomes: A systematic review and meta‐analysis. BMJ Global Health, 7(4), e008233. 10.1136/bmjgh-2021-008233 PMC8977747 35365481

[mcn13487-bib-0046] Mekonnen, T. , Dune, T. , & Perz, J. (2019). Maternal health service utilisation of adolescent women in sub‐Saharan Africa: A systematic scoping review. BMC Pregnancy and Childbirth, 19(1), 366. 10.1186/s12884-019-2501-6 31638927 PMC6805384

[mcn13487-bib-0047] Miller, L. C. , Neupane, S. , Joshi, N. , Shrestha, M. , Neupane, S. , Lohani, M. , & Thorne‐Lyman, A. L. (2020). Diet quality over time is associated with better development in rural Nepali children. Maternal & Child Nutrition, 16(3), e12964. 10.1111/mcn.12964 32048475 PMC7296824

[mcn13487-bib-0048] Moursi, M. M. , Arimond, M. , Dewey, K. G. , Trèche, S. , Ruel, M. T. , & Delpeuch, F. (2008). Dietary diversity is a good predictor of the micronutrient density of the diet of 6‐ to 23‐month‐old children in Madagascar. The Journal of Nutrition, 138(12), 2448–2453. 10.3945/jn.108.093971 19022971

[mcn13487-bib-0049] Mura Paroche, M. , Caton, S. J. , Vereijken, C. M. J. L. , Weenen, H. , & Houston‐Price, C. (2017). How infants and young children learn about food: A systematic review. Frontiers in Psychology, 8, 1046. 28790935 10.3389/fpsyg.2017.01046 PMC5524770

[mcn13487-bib-0050] Na, M. , Aguayo, V. M. , Arimond, M. , Mustaphi, P. , & Stewart, C. P. (2018). Predictors of complementary feeding practices in Afghanistan: Analysis of the 2015 Demographic and Health Survey. Maternal & child nutrition, 14(Suppl 4), e12696. 10.1111/mcn.12696 30499256 PMC6587761

[mcn13487-bib-0051] Nkoka, O. , Mhone, T. G. , & Ntenda, P. A. M. (2018). Factors associated with complementary feeding practices among children aged 6‐23 mo in Malawi: An analysis of the Demographic and Health Survey 2015‐2016. International Health, 10(6), 466–479. 10.1093/inthealth/ihy047 30052967

[mcn13487-bib-0053] OECD/FAO . (2016). OECDFAO agricultural outlook 20162025: Special focus: Sub‐Saharan Africa. OECD Publishing.

[mcn13487-bib-0054] Pelto, G. H. , Zhang, Y. , & Habicht, J. P. (2010). Premastication: The second arm of infant and young child feeding for health and survival? Maternal & Child Nutrition, 6(1), 4–18. 10.1111/j.1740-8709.2009.00200.x 20073131 PMC6860819

[mcn13487-bib-0056] Prado, E. L. , Abbeddou, S. , Adu‐Afarwuah, S. , Arimond, M. , Ashorn, P. , Ashorn, U. , Bendabenda, J. , Brown, K. H. , Hess, S. Y. , Kortekangas, E. , Lartey, A. , Maleta, K. , Oaks, B. M. , Ocansey, E. , Okronipa, H. , Ouédraogo, J. B. , Pulakka, A. , Somé, J. W. , Stewart, C. P. , … Dewey, K. G. (2017). Predictors and pathways of language and motor development in four prospective cohorts of young children in Ghana, Malawi, and Burkina Faso. Journal of Child Psychology and Psychiatry, 58(11), 1264–1275. 10.1111/jcpp.12751 28543426 PMC5697619

[mcn13487-bib-0058] Ruel, M. T. (2003). Is dietary diversity an indicator of food security or dietary quality? A review of measurement issues and research needs. Food and Nutrition Bulletin, 24(2), 231–232. 10.1177/156482650302400210 12891828

[mcn13487-bib-0059] Ruel, M. T. , Quisumbing, A. R. , & Balagamwala, M. (2018). Nutrition‐sensitive agriculture: What have we learned so far? Global Food Security, 17, 128–153.

[mcn13487-bib-0060] Rutstein, S. , & Staveteig, S. (2014). Making the Demographic and Health Surveys Wealth Index Comparable. DHS Methodological Reports No. 9. ICF International.

[mcn13487-bib-0061] Ryckman, T. , Beal, T. , Nordhagen, S. , Chimanya, K. , & Matji, J. (2021). Affordability of nutritious foods for complementary feeding in Eastern and Southern Africa. Nutrition Reviews, 79(Suppl 1), 35–51. 10.1093/nutrit/nuaa137 33693913 PMC7948081

[mcn13487-bib-0063] Sanghvi, T. , Martin, L. , Hajeebhoy, N. , Abrha, T. H. , Abebe, Y. , Haque, R. , Tran, H. T. T. , & Roy, S. (2013). Strengthening systems to support mothers in infant and young child feeding at scale. Food and Nutrition Bulletin, 34(3 Suppl), S156–S168. 10.1177/15648265130343S203 24261074

[mcn13487-bib-0065] Sibhatu, K. T. , & Qaim, M. (2018). Review: Meta‐analysis of the association between production diversity, diets, and nutrition in smallholder farm households. Food Policy, 77, 1–18.

[mcn13487-bib-0066] Solomon, D. , Aderaw, Z. , & Tegegne, T. K. (2017). Minimum dietary diversity and associated factors among children aged 6‐23 months in Addis Ababa, Ethiopia. International Journal for Equity in Health, 16(1), 181. 10.1186/s12939-017-0680-1 29025434 PMC5639776

[mcn13487-bib-0067] Southern African Development Community . (2019). Joint meeting of the SADC ministers of health and ministers responsible for HIV and AIDS. 10 November 2019 Tanzania. Record of the Meeting. SADC.

[mcn13487-bib-0068] Stewart, C. P. , Iannotti, L. , Dewey, K. G. , Michaelsen, K. F. , & Onyango, A. W. (2013). Contextualising complementary feeding in a broader framework for stunting prevention. Maternal & Child Nutrition, 9(Suppl 2), 27–45. 10.1111/mcn.12088 24074316 PMC6860787

[mcn13487-bib-0069] UNICEF . (2019). The State of the World's Children 2019: Children, Food and Nutrition: Growing Well in a Changing World.

[mcn13487-bib-0070] UNICEF . (2021). *Fed to Fail? The Crisis of Children's Diets in Early Life. 2021 Child Nutrition Report*. UNICEF.

[mcn13487-bib-0072] White, J. M. , Beal, T. , Arsenault, J. E. , Okronipa, H. , Hinnouho, G. M. , Chimanya, K. , Matji, J. , & Garg, A. (2021). Micronutrient gaps during the complementary feeding period in 6 countries in Eastern and Southern Africa: A comprehensive nutrient gap assessment. Nutrition Reviews, 79(Suppl 1), 16–25. 10.1093/nutrit/nuaa142 PMC7947982 33693910

[mcn13487-bib-0074] Working Group on Infant and Young Child Feeding Indicators . (2006). *Developing and Validating Simple Indicators of Dietary Quality and Energy Intake of Infants and Young Children in Developing Countries: Summary of findings from analysis of 10 data sets*.

[mcn13487-bib-0075] World Health Organization . (2003). Complementary feeding: Report of the global consultation, and summary of guiding principles for complementary feeding of the breastfed child.

[mcn13487-bib-0076] World Health Organization, & United Nations Children's Fund . (2017). *Global nutrition monitoring framework: Operational guidance for tracking progress in meeting targets for 2025*.

[mcn13487-bib-0078] Zimbabwe National Statistics Agency . (2019). *Zimbabwe Smallholder Agricultural Productivity Survey 2017 Report*. Retrieved from Harare, Zimbabwe

