# Supplementary material for: Factors associated with diet diversity among infants and young children in the Eastern and Southern Africa region
Source: Matern Child Nutr. 2023 Mar 15;19(3):e13487. doi: 10.1111/mcn.13487 (PMC10262889; doi:10.1111/mcn.13487)
Supplement: Supplementary file 1 — Supporting information. [file MCN-19-e13487-s001.docx]

Supplemental Table 1. List of potential factors related to child’s minimum dietary diversity

| Level | Variables reviewed at initial stage |
| --- | --- |
| Household  (n=9) | Residence (urban/rural)  Wealth quintiles  Sex of household head  Number of household members  Number of children under five years old in the household  Use of improved drinking water sources  Use of improved sanitation facilities  Time to access water sources  Type of cooking fuel |
| Maternal  (n=29) | Age (15-24y; 25-34y; 35-49y)  Highest education level  Literacy  Occupation (agriculture, not-working, non-agriculture),  BMI (<18.5; 18.5-25.0; ≥25.0)  Stunting  Currently pregnant  Smoke cigarettes  Currently breastfeeding  Currently residing with husband/partner  Number of antenatal visits during last pregnancy,  Places of the recent delivery  C-section  Put the baby to the breast withing 1 hr after birth  Delivery assisted by health professional  Postnatal check-up on child within two days after delivery  Safe disposal of child’s stool  Watching TV at least once a week  Reading newspaper at least once a week  Listening to radio at least once a week  Exposure to any media (TV, newspaper, or radio) at least once a week  Women empowerment  Tolerance of Domestic violence  Experience of child death  Decision making on household purchases for daily needs  Decision making on family visit  Decision making on respondent's health care  Decision making on large household purchase  Decision making on food to be cooked each day |
| Child  (n=10) | Sex  Age (0-5 mo, 6-23 mo, and 24-59 mo)  Birth order (1^st^, 2^nd^- 4^th^, or ≥5^th^)  Birth interval  Being breastfed within 1 hour after birth  Episodes of diarrhea in the past two weeks  Episodes of cough in the past two weeks  Episodes of fever in the past two weeks  Anemia  Vitamin-A supplementation in the past six months. |
| Community  (n=10) | Proportion of women who had c-section  Proportion of women who had 4 or higher antenatal visit  Proportion of women who had iron supplement during pregnancy  Proportion of children having iron pill  Proportion of women delivery assisted by trained health professionals  Proportion of women who had timely postnatal care  Proportion of households using improved toilet facilities  Proportion of women who completed primary or higher education within community  Proportion of “empowered women” within community  Proportion of women within community who gave birth at health facilities |

**Supplemental Table 2. Justification of variable selection**

| **Child characteristics** | **Justification** |
| --- | --- |
| Age (0-5 mo, 6-23 mo, or 24-59 mo); | Children consume more diverse and solid foods as he/she grows (Sebayang et al., 2020). |
| Birth order (1^st^, 2^nd^- 4^th^, or ≥5^th^) | Birth order is related to the competition of foods among siblings within a household (Potter et al., 2021). |
| Episodes of diarrhea/cough/fever in the past two weeks | Sick children lose appetite and may refuse to consume sufficient and diverse foods. Some local taboos prevent mothers giving diverse foods or specific foods (e.g., milk) to sick children (Freitas, Albuquerque, Silva, & Oliveira, 2018). |
| Vitamin-A supplementation in the past six months | The children who did not receive annual vitamin A supplementation may not access to other health services from near health providers (Sagalova, Zagre, & Vollmer, 2020). |
| **Maternal characteristics** |  |
| Age (15-24y, 25-34y, or 35-49y) | Adolescent and young mothers may not have appropriate knowledge and practices on appropriate child feeding and caring and family planning and their needs for additional education remain unmet peer mothers (Hackett, Mukta, Jalal, & Sellen, 2015). |
| Highest education level | Many studies reported maternal education is as a determinant of meeting optimal complementary feeding practices including minimum dietary diversity (Khanal, Sauer, & Zhao, 2013) |
| occupation (employment in agriculture, no formal occupation, waged occupation) | Mother’s occupation is related to the hours of daily labor, amount of income, and capacity of access to diverse foods. |
| Body Mass Index (BMI) (<18.5; 18.5-25.0; ≥25.0 kg/m^2^) | Mother’s nutrition status is associated with child growth, genetically and physically (Martorell & Zongrone, 2012). |
| Antenatal visits during last pregnancy | Frequent antenatal visit increases the chance of contacting health providers and receiving more comprehensive nutrition counseling and advice about breastfeeding, complementary feeding and childcare (Sebayang et al., 2020; Young & Kang, 2021) |
| Places of recent delivery | Delivery at health centers/hospitals is associated with safe health and increase of contacting health providers. Home delivery may not prevent or treat adverse event during delivery |
| Delivery assisted by health professional | Delivery with trained health professionals is associated with safe health and increase of having optimal nutrition counseling. Mothers with home delivery may not prevent or treat adverse events during delivery (Young & Kang, 2021) |
| Watching TV at least once a week | Frequent exposure to media help mothers to have appropriate knowledge on child’s health care and increased access to resources for childcare (Walters, Rakotomanana, Komakech, & Stoecker, 2019). |
| Reading a newspaper at least once a week | Frequent exposure to media help mothers to have appropriate knowledge on child’s health care and increased access to resources for childcare (Walters et al., 2019) |
| Listening to radio at least once a week, exposure to any media (TV, newspaper, or radio) at least once a week | Frequent exposure to media help mothers to have appropriate knowledge on child’s health care and increased access to resources for childcare (Walters et al., 2019) |
| Women’s empowerment | Women empowerment was generated for each country using established methods, based on two domains of maternal participation in decision making and overall attitude toward domestic violence (Jennings et al., 2014). |
| **Household characteristics** |  |
| Wealth status | Higher wealth status relates to household food security and provides more opportunities to purchase various foods (Agbadi, Urke, & Mittelmark, 2017) |
| Rural/urban | Urban markets provide diversified and processed food choices than rural areas (Codjoe, Okutu, & Abu, 2016) |
| No of children <5 y living in the same household | Relate the competition of foods within household. (Sebayang et al., 2020). |
| Sex of the head of household | Children living in mother-headed households often use unprotected sources of water  (Workie, Mekonen, Fekadu, & Mekonen, 2020). Children in female headed households may experience low dietary diversity (Ochieng, Afari-Sefa, Lukumay, & Dubois, 2017) |
| Improved drinking water sources | Improved drinking water sources was associated with child stunting and child anemia (Kang & Kim, 2019). |
| Improved toilet facility | suboptimal household water access influence food choice and prevents crop diversification (Choudhury et al., 2019) |
| **Community-level variables** |  |
| Proportion of women who completed primary or higher education within community | Previous studies reported that community level education as well as individual education relates to the child’s dietary diversity (Na et al., 2018) |
| Proportion of women within community who gave birth at health facilities | Community members may use the same health service and health facilities. Community mothers may have the same nutrition and health counseling messages from health providers (Na et al., 2018) |
| Proportion of “empowered women” within community. | Some literature reported that women empowerment at community level relates to the child’s diet (Na et al., 2018) |

**Supplemental Table 3. Absolute difference in MDD between richest and poorest wealth quintiles and rural and urban residence^1,2^**

| Country | Richest (%) | Poorest (%) | wealth gap (%pt) | Urban (%) | Rural (%) | Area gap (%pt) |
| --- | --- | --- | --- | --- | --- | --- |
| ***MDD*** |  |  |  |  |  |  |
| Ethiopia | 24.9 | 6.2 | 18.7*** | 27.3 | 9.9 | 17.4*** |
| Kenya | 57.3 | 17.3 | 40.0*** | 49.9 | 27.4 | 22.5*** |
| Malawi | 40.4 | 13.9 | 26.5*** | 37.9 | 19.8 | 18.1*** |
| Rwanda | 45.1 | 14.6 | 30.5*** | 43.1 | 24.3 | 18.8*** |
| South Africa | 50.6 | 36.2 | 14.4 | 42.6 | 32.1 | 10.5** |
| Tanzania | 39.1 | 12.1 | 27.0*** | 31.4 | 16.7 | 14.7*** |
| Uganda | 33.1 | 17.9 | 15.2*** | 28.3 | 22.8 | 5.5* |
| Zambia | 32.6 | 13.6 | 19.0*** | 24.1 | 15.1 | 9.0*** |
| Zimbabwe | 43.0 | 11.9 | 31.1*** | 36.1 | 16.0 | 20.1*** |
| ***Grain*** |  |  |  |  |  |  |
| Ethiopia | 75.5 | 55.5 | 20.0** | 73.1 | 56.5 | 16.6*** |
| Kenya | 90.6 | 76.6 | 14.0*** | 81.8 | 79.1 | 2.7** |
| Malawi | 77.9 | 63.1 | 14.8*** | 78.6 | 68.8 | 9.9** |
| Rwanda | 76.3 | 65.6 | 10.7** | 76.1 | 68.9 | 7.2*** |
| South Africa | 91.3 | 83.9 | 7.4 | 86.7 | 87.1 | -0.4 |
| Tanzania | 91.4 | 83.7 | 7.8*** | 92.0 | 87.9 | 4.1* |
| Uganda | 84.2 | 77.0 | 7.2* | 83.7 | 79.7 | 4.0** |
| Zambia | 86.9 | 81.0 | 5.9*** | 83.3 | 76.1 | 7.2*** |
| Zimbabwe | 96.9 | 90.7 | 6.1 | 94.5 | 91.2 | 3.3 |
| ***Beans*** |  |  |  |  |  |  |
| Ethiopia | 31.3 | 13.9 | 17.3** | 22.7 | 16.1 | 6.6*** |
| Kenya | 28.2 | 20.0 | 8.3** | 23.6 | 21.5 | 2.2 |
| Malawi | 34.9 | 18.6 | 16.3*** | 27.4 | 24.0 | 3.3 |
| Rwanda | 57.8 | 63.0 | -5.2** | 60.2 | 66.6 | -6.4* |
| South Africa | 11.1 | 18.6 | -7.5 | 12.8 | 17.6 | -4.8 |
| Tanzania | 40.8 | 28.2 | 12.5*** | 38.9 | 32.3 | 6.6* |
| Uganda | 49.4 | 44.2 | 5.1 | 49.0 | 47.7 | 1.3 |
| Zambia | 23.0 | 17.5 | 5.5 | 21.6 | 17.1 | 4.5 |
| Zimbabwe | 27.6 | 16.1 | 11.6* | 21.5 | 17.4 | 4.1* |
| ***Flesh*** |  |  |  |  |  |  |
| Ethiopia | 17.0 | 5.6 | 11.4*** | 17.5 | 6.6 | 10.9 |
| Kenya | 29.8 | 16.3 | 13.6** | 26.1 | 17.6 | 8.5*** |
| Malawi | 43.9 | 22.4 | 21.5*** | 42.8 | 31.1 | 11.7*** |
| Rwanda | 29.3 | 12.2 | 17.1*** | 22.7 | 16.6 | 6.1*** |
| South Africa | 44.9 | 48.1 | -3.2 | 47.8 | 47.4 | 0.4 |
| Tanzania | 53.4 | 20.8 | 32.7*** | 47.5 | 31.2 | 16.3*** |
| Uganda | 41.1 | 31.6 | 9.5*** | 37.4 | 31.8 | 5.6** |
| Zambia | 54.7 | 30.7 | 24.0*** | 46.3 | 36.3 | 10.0*** |
| Zimbabwe | 59.6 | 29.3 | 30.3*** | 56.0 | 33.7 | 22.3*** |
| ***Vitamin A-rich fruits and vegetables*** |  |  |  |  |  |  |
| Ethiopia | 38.9 | 19.4 | 19.6** | 40.0 | 22.1 | 18.0*** |
| Kenya | 83.2 | 41.9 | 41.2*** | 65.5 | 50.9 | 14.6*** |
| Malawi | 74.4 | 71.9 | 2.5 | 72.6 | 74.1 | -1.5 |
| Rwanda | 72.6 | 61.7 | 10.9*** | 73.9 | 68.8 | 5.0* |
| South Africa | 66.0 | 41.8 | 24.1* | 56.2 | 41.0 | 15.2** |
| Tanzania | 72.3 | 59.5 | 12.8** | 68.8 | 61.5 | 7.3** |
| Uganda | 41.2 | 56.4 | -15.2*** | 42.4 | 51.7 | -9.3*** |
| Zambia | 62.3 | 60.4 | 2.0 | 58.7 | 61.1 | -2.4 |
| Zimbabwe | 49.9 | 63.3 | -13.3* | 49.2 | 58.0 | -8.8*** |
| ***Other fruits and vegetables*** |  |  |  |  |  |  |
| Ethiopia | 19.9 | 5.8 | 14.0*** | 20.6 | 7.8 | 12.7*** |
| Kenya | 55.1 | 15.8 | 39.3*** | 36.6 | 22.4 | 14.2*** |
| Malawi | 34.9 | 23.0 | 12.0**** | 32.9 | 28.3 | 4.6 |
| Rwanda | 36.4 | 17.1 | 19.2*** | 35.2 | 23.1 | 12.2*** |
| South Africa | 49.3 | 31.3 | 18.0 | 45.6 | 36.2 | 9.4 |
| Tanzania | 32.8 | 15.2 | 17.6*** | 27.6 | 17.9 | 9.7*** |
| Uganda | 26.9 | 15.0 | 11.9*** | 22.3 | 18.2 | 4.1* |
| Zambia | 26.4 | 16.6 | 9.8*** | 21.3 | 16.6 | 4.7** |
| Zimbabwe | 40.5 | 22.2 | 18.4*** | 36.3 | 21.6 | 14.7*** |
| ***Eggs*** |  |  |  |  |  |  |
| Ethiopia | 29.0 | 10.7 | 18.2*** | 26.4 | 13.3 | 13.1*** |
| Kenya | 24.0 | 12.2 | 11.9*** | 17.7 | 14.7 | 3.0* |
| Malawi | 20.3 | 6.8 | 13.6*** | 18.9 | 11.4 | 7.5*** |
| Rwanda | 11.6 | 1.1 | 10.5*** | 7.2 | 3.2 | 4.0*** |
| South Africa | 54.5 | 41.3 | 13.2 | 42.3 | 37.6 | 4.6 |
| Tanzania | 18.9 | 3.0 | 15.8*** | 11.2 | 5.5 | 5.7*** |
| Uganda | 25.2 | 8.9 | 16.3*** | 19.2 | 11.3 | 7.9*** |
| Zambia | 32.8 | 9.7 | 23.2*** | 24.7 | 14.2 | 10.5*** |
| Zimbabwe | 31.4 | 8.7 | 22.7*** | 25.2 | 11.0 | 14.2*** |
| ***Dairy*** |  |  |  |  |  |  |
| Ethiopia | 52.6 | 39.3 | 13.3** | 54.6 | 40.2 | 14.4*** |
| Kenya | 71.6 | 49.5 | 22.2*** | 62.8 | 56.5 | 6.3*** |
| Malawi | 34.8 | 5.2 | 29.5*** | 26.7 | 8.1 | 18.7*** |
| Rwanda | 47.5 | 12.2 | 35.3*** | 32.6 | 18.9 | 13.7*** |
| South Africa | 89.4 | 62.7 | 26.7*** | 78.1 | 62.4 | 15.7*** |
| Tanzania | 34.5 | 26.5 | 8.0*** | 24.7 | 20.7 | 4.0 |
| Uganda | 51.7 | 13.8 | 37.9*** | 45.9 | 23.9 | 22.0*** |
| Zambia | 31.9 | 3.7 | 28.3*** | 16.5 | 5.4 | 11.1*** |
| Zimbabwe | 49.7 | 9.9 | 39.8*** | 41.0 | 14.2 | 26.8*** |
| ***Breastmilk*** |  |  |  |  |  |  |
| Ethiopia | 85.6 | 82.2 | 3.3 | 77.0 | 81.8 | -4.8 |
| Kenya | 76.5 | 80.2 | -3.7 | 78.6 | 80.4 | -1.8 |
| Malawi | 80.0 | 87.3 | -7.3** | 81.4 | 84.3 | -3.0 |
| Rwanda | 83.1 | 93.5 | -10.4*** | 85.2 | 93.2 | -8.0*** |
| South Africa | 30.8 | 55.3 | -24.5* | 45.8 | 45.7 | 0.1 |
| Tanzania | 74.9 | 80.3 | -5.3 | 73.1 | 78.5 | -5.4** |
| Uganda | 62.4 | 80.0 | -17.6*** | 66.2 | 76.1 | -9.9*** |
| Zambia | 63.0 | 90.6 | -27.6*** | 72.4 | 85.6 | -13.2*** |
| Zimbabwe | 60.8 | 73.7 | -12.9* | 63.0 | 72.1 | -9.1*** |

MDD, minimum dietary diversity; pt, percentage point

*p<0.05; ** p<0.01; ***p<0.001

^1^ All values (weighted %) were estimated accounted for survey design and sampling weights

^2^ Positive numbers in wealth gap (%pt) and Area gap (%pt) indicate that wealthier households have a greater prevalence of attaining MDD (Panel A), and that urban households have a greater prevalence of attaining MDD (Panel B), and negative numbers indicate the opposite.

**Supplemental Table 3. Household, parental, and child characteristics among children 6-23 months of age in 9 Eastern and Southern African countries^1^**

| Characteristics | Ethiopia  (n=2965) | Kenya (n=2906) | Malawi  (n=4879) | Rwanda  (n=2430) | South Africa (n=872) | Tanzania  (n=3170) | Uganda  (4418) | Zambia  (n=3776) | Zimbabwe  (n=1656) |
| --- | --- | --- | --- | --- | --- | --- | --- | --- | --- |
|  | Weighted % | Weighted % | Weighted % | Weighted % | Weighted % | Weighted % | Weighted % | Weighted % | Weighted % |
| *Household level* |  |  |  |  |  |  |  |  |  |
| Area |  |  |  |  |  |  |  |  |  |
| Urban | 12.2 | 35.9 | 13.4 | 16.7 | 61.3 | 27.5 | 22.2 | 33.5 | 27.3 |
| Rural | 87.8 | 64.1 | 86.6 | 83.3 | 38.7 | 72.5 | 77.8 | 66.5 | 72.7 |
| Wealth quintiles |  |  |  |  |  |  |  |  |  |
| Highest | 15.2 | 19.7 | 15.5 | 17.4 | 12.6 | 17.3 | 20.1 | 14.4 | 14.4 |
| High | 18.4 | 18.5 | 16.6 | 17.3 | 19.9 | 18.9 | 17.2 | 17.0 | 21.4 |
| Middle | 22.2 | 17.1 | 19.3 | 19.4 | 22.0 | 18.6 | 19.1 | 20.7 | 17.8 |
| Low | 21.2 | 19.7 | 23.0 | 21.2 | 22.6 | 21.3 | 20.8 | 22.6 | 20.1 |
| Lowest | 23.1 | 25.0 | 25.6 | 24.6 | 22.9 | 23.9 | 22.8 | 25.3 | 26.4 |
| Sex of HH |  |  |  |  |  |  |  |  |  |
| Male | 86.5 | 71.0 | 75.3 | 79.6 | 47.3 | 82.9 | 76.5 | 81.6 | 62.7 |
| Female | 13.5 | 29.0 | 24.7 | 20.4 | 52.8 | 17.2 | 23.5 | 18.4 | 37.3 |
| No of children <5 y living in the same household |  |  |  |  |  |  |  |  |  |
| 1 | 38.4 | 41.6 | 46.2 | 47.1 | 53.3 | 37.2 | 33.8 | 32.3 | 46.1 |
| ≥2 or more | 61.6 | 58.4 | 53.8 | 52.9 | 46.7 | 62.8 | 66.2 | 67.7 | 53.9 |
| Improved drinking water source^2^ | 57.0 | 64.7 | 85.5 | 70.6 | 86.2 | 53.2 | 75.5 | 57.6 | 72.9 |
| Improved toilet facility | 10.4 | 46.3 | 81.6 | 30.9 | 69.9 | 26.8 | 33.0 | 38.7 | 56.5 |
| Time to get to water source (min) |  |  |  |  |  |  |  |  |  |
| Inside the house | 12.1 | 28.6 | 12.4 | 8.8 | 73.2 | 22.0 | 14.9 | 19.5 | 30.6 |
| 1-59 min | 58.5 | 53.5 | 63.4 | 69.2 | 19.0 | 51.9 | 55.9 | 67.9 | 51.4 |
| ≥ 60 min | 29.4 | 19.9 | 24.2 | 22.0 | 7.8 | 26.1 | 29.2 | 12.6 | 18.0 |
| Type of cooking fuel^3^ |  |  |  |  |  |  |  |  |  |
| Efficient | 3.4 | 15.4 | 1.2 | 0.3 | 78.4 | 1.2 | 0.4 | 7.7 | 23.3 |
| Inefficient | 96.6 | 84.7 | 98.8 | 99.7 | 21.6 | 98.8 | 99.6 | 92.3 | 76.7 |
|  |  |  |  |  |  |  |  |  |  |
| *Maternal level* |  |  |  |  |  |  |  |  |  |
| Maternal age (years) |  |  |  |  |  |  |  |  |  |
| 15-24 | 27.5 | 36.5 | 44.0 | 25.3 | 34.4 | 37.3 | 41.0 | 37.2 | 37.1 |
| 25-34 | 52.2 | 49.8 | 39.8 | 51.9 | 49.0 | 43.1 | 42.9 | 44.1 | 48.2 |
| 35-49 | 20.3 | 13.8 | 16.1 | 22.8 | 16.7 | 19.6 | 16.1 | 18.8 | 14.7 |
| Maternal education |  |  |  |  |  |  |  |  |  |
| Higher | 2.9 | 7.7 | 1.9 | 2.2 | 10.9 | 1.0 | 7.3 | 3.8 | 4.0 |
| Secondary | 5.5 | 26.6 | 19.1 | 11.8 | 80.5 | 16.2 | 23.0 | 30.5 | 61.5 |
| Primary | 30.7 | 53.3 | 67.3 | 71.9 | 7.8 | 62.9 | 60.4 | 55.3 | 33.0 |
| No formal education | 60.9 | 12.4 | 11.7 | 14.1 | 0.7 | 19.9 | 9.4 | 10.5 | 1.4 |
| Occupation |  |  |  |  |  |  |  |  |  |
| Agriculture | 21.3 | 23.6 | 44.6 | 75.0 | 1.4 | 55.5 | 44.1 | 33.9 | 13.2 |
| No formal work | 57.4 | 40.6 | 30.6 | 7.1 | 71.2 | 18.1 | 19.0 | 43.1 | 51.0 |
| Waged job | 21.3 | 35.8 | 24.8 | 17.9 | 27.4 | 26.4 | 3.7 | 22.9 | 35.8 |
| Body mass index (kg/m^2^) |  |  |  |  |  |  |  |  |  |
| <18.5 | 24.0 | 9.6 | 5.1 | 4.7 | 3.6 | 7.9 | 9.5 | 9.6 | 5.6 |
| 18.5-24.9 | 69.9 | 62.6 | 80.9 | 72.8 | 37.0 | 70.5 | 68.4 | 71.4 | 67.0 |
| ≥25 | 6.1 | 27.8 | 14.0 | 22.5 | 59.4 | 21.6 | 22.1 | 18.9 | 27.4 |
| Number of antenatal clinic visits |  |  |  |  |  |  |  |  |  |
| ≥4 | 37.8 | 57.1 | 51.0 | 46.0 | 76.7 | 52.3 | 64.6 | 57.2 | 77.4 |
| 0-3 | 62.2 | 42.9 | 49.0 | 54.0 | 23.3 | 47.7 | 35.4 | 42.8 | 22.7 |
| Place of delivery |  |  |  |  |  |  |  |  |  |
| Home | 63.1 | 35.5 | 7.0 | 8.2 | 3.9 | 35.4 | 23.7 | 28.2 | 19.8 |
| Health facility | 36.9 | 54.5 | 93.0 | 91.8 | 96.1 | 64.6 | 76.3 | 71.8 | 80.2 |
| Type of delivery assistance |  |  |  |  |  |  |  |  |  |
| Non-professionals^4^ | 62.2 | 34.7 | 8.5 | 5.8 | 3.3 | 85.7 | 22.0 | 31.7 | 18.6 |
| Professionals | 37.8 | 65.3 | 91.5 | 94.2 | 96.7 | 14.3 | 78.0 | 68.3 | 81.4 |
| Exposure to media at least once a week^5^ | 19.5 | 71.9 | 33.2 | 57.0 | 80.4 | 47.5 | 61.9 | 57.5 | 48.2 |
| Reading newspaper at least once a week | 1.7 | 12.1 | 5.7 | 3.0 | 38.1 | 9.7 | 7.6 | 14.5 | 10.7 |
| Listening to radio at least once a week | 14.2 | 64.9 | 27.8 | 55.2 | 49.9 | 40.9 | 57.1 | 47.5 | 33.2 |
| Watching TV at least once a week | 9.5 | 31.5 | 7.5 | 10.8 | 71.2 | 19.1 | 16.9 | 29.5 | 24.6 |
| Women empowerment^6^ |  |  |  |  |  |  |  |  |  |
| High | 69.9 | 49.4 | 52.7 | 68.3 | 91.1 | 64.4 | 43.3 | 51.7 | 71.6 |
| Low | 30.1 | 50.6 | 47.3 | 31.7 | 8.9 | 35.6 | 56.8 | 48.3 | 28.4 |
|  |  |  |  |  |  |  |  |  |  |
| Child level |  |  |  |  |  |  |  |  |  |
| Age (months) |  |  |  |  |  |  |  |  |  |
| Mean (SE) | 14.0 (0.1) | 14.4 (0.1) | 14.4 (0.1) | 14.1 (0.1) | 14.2 (0.2) | 14.5 (0.1) | 14.2 (0.1) | 14.4 (0.1) | 14.3 (0.1) |
| 6-11 | 34.6 | 33.3 | 34.2 | 36.7 | 34.1 | 32.3 | 35.9 | 33.6 | 33.0 |
| 12-17 | 36.4 | 34.6 | 33.7 | 32.4 | 35.6 | 35.7 | 31.9 | 35.0 | 36.4 |
| 18-23 | 29.1 | 32.1 | 32.1 | 30.8 | 30.3 | 32.0 | 32.2 | 31.5 | 30.6 |
| Birth order |  |  |  |  |  |  |  |  |  |
| 1^st^ | 19.5 | 25.5 | 26.8 | 28.5 | 33.8 | 26.2 | 22.5 | 21.3 | 25.2 |
| 2^nd^ - 4^th^ | 42.9 | 52.4 | 49.1 | 50.0 | 60.8 | 45.8 | 46.7 | 46.3 | 59.0 |
| 5^th^ or lower | 37.6 | 22.1 | 24.1 | 21.6 | 5.4 | 28.0 | 30.8 | 32.4 | 15.8 |
| Diarrhea | 19.4 | 24.9 | 38.8 | 20.3 | 18.2 | 21.4 | 34.3 | 28.0 | 30.5 |
| Cough | 24.3 | 41.1 | 27.9 | 34.2 | 30.7 | 21.1 | 48.4 | 28.0 | 41.4 |
| Fever | 20.0 | 29.8 | 35.8 | 24.3 | 25.4 | 22.5 | 39.4 | 26.8 | 17.7 |
| Vitamin A supplementation in the past 6 months | 57.3 | 76.3 | 65.6 | 80.5 | 75.6 | 43.5 | 62.8 | 68.8 | 72.2 |

^1^ All values (weighted %) are estimated accounted for survey design and sampling weights

^2^ Improved drinking water sources piped into dwelling piped to yard/plot, public tap/standpipe, piped to neighbor, tube well or borehole, protected well, protected spring, rainwater

^3^ Efficient cooking fuel includes electricity, gas, and LPG while inefficient ones include charcoal, animal dung, wood, and others.

^4^ Health extension workers, health officer, traditional birth attendant, family/friends/neighbor

^5^ Watching tv, reading a newspaper, or listening to the radio at least once a week

^6^ Women's empowerment score: Total number of "yes" of questions under decision-making domain, plus 1 if "high" or plus 0 if "low" in overall attitude toward domestic

**Supplemental Table 4.** Comparison seven-group minimum dietary diversity (MDD7)^1^ and eight-group minimum dietary diversity (MDD8) by wealth quintiles among nine countries

| Characteristics | Ethiopia | | Kenya (n=2906) | | Malawi | | Rwanda | | South Africa (n=872) | | Tanzania | | Uganda | | Zambia | | Zimbabwe | |
| --- | --- | --- | --- | --- | --- | --- | --- | --- | --- | --- | --- | --- | --- | --- | --- | --- | --- | --- |
|  | (n=2965) | |  |  | (n=4879) | | (n=2430) | |  |  | (n=3170) | | (n=4418) | | (n=3776) | | (n=1656) | |
| Wealth quintile | ^1^MDD7 | ^1^MDD8 | ^2^MDD7 | ^2^MDD8 | ^3^MDD7 | 3MDD8 | ^4^MDD7 | MDD8 | ^5^MDD7 | MDD8 | ^6^MDD7 | MDD8 | ^7^MDD7 | MDD8 | ^8^MDD7 | MDD8 | ^9^MDD7 | MDD8 |
|  | ^Weighted %^ | ^Weighted %^ | ^Weighted %^ | ^Weighted %^ | ^Weighted %^ | ^Weighted %^ | ^Weighted %^ | ^Weighted %^ | ^Weighted %^ | ^Weighted %^ | ^Weighted %^ | ^Weighted %^ | ^Weighted %^ | ^Weighted %^ | ^Weighted %^ | ^Weighted %^ | ^Weighted %^ | ^Weighted %^ |
| 1^st^ | 7.2 | 6.2 | 20.2 | 17.2 | 15.4 | 13.9 | 16.5 | 14.6 | 45.4 | 36.2 | 14.3 | 12.1 | 20.8 | 17.9 | 15.1 | 13.6 | 17.1 | 11.9 |
| 2^nd^ | 11.2 | 9.6 | 29.4 | 24.9 | 20.9 | 18.8 | 22.1 | 21.1 | 41.4 | 33.0 | 17.7 | 12.4 | 26.9 | 22.8 | 17.6 | 15.4 | 19.4 | 16.5 |
| 3^rd^ | 11.5 | 11.2 | 39.6 | 32.2 | 23.1 | 21.1 | 26.2 | 23.8 | 42.7 | 37.1 | 22.9 | 18.1 | 25.6 | 22.0 | 18.2 | 15.3 | 24.2 | 17.6 |
| 4^th^ | 14.8 | 12.2 | 55.0 | 50.5 | 27.6 | 24.2 | 40.7 | 39.7 | 52.9 | 41.3 | 30.8 | 26.6 | 30.4 | 25.0 | 24.6 | 19.7 | 36.6 | 26.7 |
| 5^th^ | 27.2 | 24.9 | 62.8 | 57.6 | 44.3 | 40.4 | 49.1 | 45.1 | 61.2 | 50.6 | 48.4 | 39.1 | 40.3 | 33.1 | 41.8 | 32.6 | 53.8 | 43.0 |
| Total | 13.4 | 12.0 | 40.1 | 35.4 | 24.7 | 22.2 | 29.5 | 27.4 | 47.4 | 38.5 | 25.6 | 20.7 | 28.6 | 24.0 | 21.8 | 18.2 | 28.2 | 21.5 |
| Wealth gap (Difference between 5^th^ quintile-1^st^ quintile) | 6.2 | 5.8 | 19.9 | 18.2 | 9.3 | 8.3 | 13 | 12.8 | 2 | 2.3 | 11.3 | 8.6 | 7.8 | 6.1 | 6.7 | 4.6 | 11.1 | 9.6 |

^1^ Seven group Minimum Dietary diversity (MDD) was calculated using Ethiopia DHS 2016

^2^ All values were estimated accounted for survey design and sampling weights.

**Supplemental Table 5**. Multivariable models of the odds of not reaching minimum dietary diversity^1^ in 9 Eastern and Southern African countries (Variables included in multivariable models in Figure 4)

| Characteristics | Ethiopia  OR (95%CI)  (n=2965) | Kenya OR (95%CI) (n=2906) | Malawi OR (95%CI) (n=4879) | Rwanda  OR (95%CI) (n=2430) | South Africa OR (95%CI) (n=872) | Tanzania  OR (95%CI) (n=3170) | Uganda OR (95%CI) (n=4418) | Zambia OR (95%CI) (n=3776) | Zimbabwe OR (95%CI) (n=1656) |
| --- | --- | --- | --- | --- | --- | --- | --- | --- | --- |
| ***Household Characteristics*** |  |  |  |  |  |  |  |  |  |
| Rural (Ref: Urban) | 1.11  (0.39, 3.15) | 1.11  (0.83, 1.48) | 1.15  (0.77,1.70) | 1.50  (1.10, 2.06)*^2^ | 1.37  (0.95, 1.98) | 0.96  (0.68, 1.33) | 0.84  (0.60, 1.19) | 1.03  (0.71, 1.49) | 1.27  (0.69, 2.33) |
| Lowest wealth quintile (Ref: Highest) | 0.94  (0.38, 2.33) | 1.56  (0.93, 2.70) | 2.13  (1.43, 3.17)* | 1.78  (1.11, 2.85)* | 0.83  (0.35, 1.96) | 2.08  (1.14, 3.78)* | 1.31 (0.87, 1.96) | 1.31  (0.71, 2.44) | 1.92 (0.81, 4.51) |
| Low wealth quintile (Ref: Highest) | 0.70  (0.38, 2.33) | 1.80 (1.11, 2.90)* | 1.68  (1.15, 2.45)* | 1.31  (0.85, 2.01) | 1.38  (0.66, 2.89) | 2.36  (1.34, 4.16)* | 1.13 (0.77, 1.66) | 1.29 (0.72, 2.31) | 1.45 (0.66, 3.20) |
| Middle wealth quintile (Ref: Highest) | 0.76  (0.34, 1.68) | 1.42 (0.89, 2.29) | 1.66  (1.15, 2.41) | 1.32  (0.86, 2.03) | 1.39  (0.66, 2.92) | 1.84  (1.05, 3.23) | 1.25 (0.87, 1.82) | 1.39 (0.81, 2.41) | 1.49 (0.66, 3.34) |
| High wealth quintile (Ref: Highest) | 0.81  (0.35, 1.88) | 0.94  (0.62, 1.45) | 1.43  (1.00, 2.04)* | 0.68  (0.46, 1.02) | 1.30  (0.67, 2.54) | 1.20  (0.81, 1.79) | 1.18  (0.82, 1.69) | 1.27  (0.83, 1.94) | 1.43  (0.92, 2.21) |
| Female household head (Ref: Male) | n/a | n/a | 1.25  (1.01, 1.54) | 1.13  (0.87, 1.47) | n/a | n/a | n/a | n/a | n/a |
| Two or more children <5 y living in the household (Ref: 1) | n/a | 0.90  (0.68, 1.18) | n/a | 1.27  (1.01, 1.60) | n/a | 1.26  (0.97, 1.64) | n/a | n/a | n/a |
| Unimproved water source (Ref: Improved) | 1.49  (0.97, 2.30) | 1.15  (0.88, 1.50) | 1.03  (0.77, 1.38) | 1.27  (0.99, 1.62) | n/a | 1.06  (0.81, 1.39) | n/a | 1.03  (0.79, 1.35) | 1.00  (0.67, 1.49) |
| Unimproved toilet (Ref: Improved) | 1.49  (0.91, 2.43) | 1.27  (0.97, 1.65) | 1.22  (0.95, 1.56) | 0.97  (0.76, 1.23) | n/a | 0.89  (0.62, 1.28) | 1.28  (1.00, 1.63) | 1.03  (0.79, 1.33) | 1.14  (0.77,1.68) |
| Time to get water source 1-<60 m (Ref: inside) | 1.57  (1.30, 5.38)* | 1.05  (0.75, 1.46) | 1.08  (0.75, 1.56) | 1.19  (0.78, 1.83) | 1.63  (0.84, 3.15) | 0.95  (0.66, 1.37) | n/a | 1.27  (0.84, 1.93) | 0.80  (0.45, 1.42) |
| Time to get to water source ≥1hr (Ref. Inside) | 2.64  (1.30, 5.38)* | 1.05  (0.75, 1.46) | 0.96  (0.69, 1.32) | 1.19  (0.78, 1.83) | 1.43  (0.80, 2.54) | 1.01  (0.73, 1.39) | n/a | 1.39 (1.03, 1.89)* | 0.83  (0.54, 1.26) |
| Non-efficient cooking fuel (Ref: Efficient) | 0.47  (0.22, 1.04) | 1.97  (1.11, 3.48)* | 2.42  (1.14, 5.13)* | 1.70  (0.44, 6.63) | n/a | n/a | 2.46 (0.72, 8.39) | 0.82 (0.50, 1.32) | 1.37  (0.78, 2.42) |
| ***Maternal Characteristics*** |  |  |  |  |  |  |  |  |  |
| Mother’s secondary education (Ref: Higher) | 2.70 (1.19, 6.10)* | 0.69  (0.41, 1.15) | 1,01  (0.47, 1.16) | 0.61  (0.31, 1.20) | n/a | 1.72  (0.75, 3.96) | 1.33  (0.93, 1.90) | 1.34 (0.75, 2.40) | 1.41  (0.76, 2.62) |
| Mother’s primary education (Ref: Higher) | 2.78  (1.19, 6.50)* | 1.08  (0.65, 1.80) | 1.11  (0.52, 2.39) | 1.06 (0.53, 2.10) | n/a | 2.40  (1.05, 5.50)* | 1.68 (1.16, 2.43)* | 1.71 (0.92, 3.18) | 1.80  (0.85, 3.81) |
| Mother’s no formal education (Ref: Higher) | 3.19  (1.37, 7.43)* | 1.31  (0.67, 2.57) | 1.84  (0.81, 4.18) | 1.36 (0.63, 2.95) | n/a | 3.00 (1.22, 7.36)* | 2.28 (1.43, 3.62)* | 1.53  (0.78, 3.01) | 2.03 (0.34, 12.2) |
| Mother’s agriculture (Ref: Waged job) | 1.20  (0.65, 2.21) | 0.85  (0.63, 1.14) | 1.53  (1.19, 1.97)* | 1.05  (0.76, 1.44) | 4.50  (0.86, 23.7) | 0.92  (0.69, 1.24) | 1.18  (0.96, 1.44) | 1.24  (0.90 1.71) | 0.84  (0.51, 1.39) |
| Mother’s no formal work (Ref: Waged job) | 1.50  (0.97, 2.31) | 1.76  (1.30, 2.38)* | 1.88  (1.45, 2.44)* | 1.86  (1.13, 3.04)* | 1.27  (0.83, 1.95) | 1.43  (0.99, 2.07) | 1.57  (1.21, 2.03)* | 1.48  (1.13, 1.94)* | 1.38 (1.02, 1.86)* |
| BMI <18.5 (Ref: 18.5-25.0) | 1.09  (0.67, 1.77) | 1.01  (0.68, 1.49) | n/a | n/a | n/a | 1.10  (0.71, 1.72) | n/a | n/a | 1.29  (0.64, 2.61) |
| BMI ≥25.0 (Ref: 18.5-25.0) | 0.79  (0.40, 1.60) | 1.09  (0.81, 1.46) | n/a | n/a | n/a | 0.88  (0.69, 1.13) | n/a | n/a | 0.75  (0.53, 1.05) |
| Antennal visits 0-3 times (Ref: 4 or more) | n/a | n/a | n/a | n/a | n/a | n/a | n/a | 1.45  (1.16, 1.80) | n/a |
| Home delivery (Ref: facility delivery) | 0.88 (0.55, 1.41) | n/a | n/a | n/a | n/a | 1.09  (0.79, 1.50) | 0.92  (0.74, 1.13) | n/a | 0.72  (0.44, 1.17) |
| No delivery assist (Ref: Yes) | n/a | 0.82  (0.59, 1.12) | n/a | n/a | n/a | n/a | n/a | 1.10  (0.83, 1.46) | n/a |
| Low women empowerment (Ref: High) | 0.59  (0.38, 0.93)* | n/a | n/a | n/a | n/a | n/a | n/a | n/a | n/a |
| Mother’s age 15-24 y (Ref: 25-34 y) | n/a | 1.18  (0.86, 1.61) | 1.35  (1.05, 1.73)* | n/a | n/a | 1.57  (1.14, 2.16)* | 1.27  (1.07, 1.50)* | 1.20 (0.93, 1.53) | 1.16  (0.86, 1.56) |
| Mother’s age 35-48 y (Ref: 25-34 y) | n/a | 0.92  (0.65, 1.31) | 1.06  (0,76, 1.48) | n/a | n/a | 1.40  (0.99, 1.97) | 0.98 (0.77, 1.25) | n/a | 1.60  (1.07, 2.39)* |
| No exposure to any media (Ref: Yes) | n/a | n/a | 1.49  (1.23, 1.82)* | 1.76  (1.38, 2.24)* | n/a | 1.84  (1.43, 2.37)* | n/a | n/a | n/a |
| No watching TV (Ref: Yes) | n/a | n/a | n/a | 1.70  (1.16, 2.49)* | 1.39  (0.92, 2.10) | n/a | n/a | n/a | 1.19  (0.82, 1.74) |
| No listening to radio (Ref: Yes) | 1.79  (1.06, 3.03)* | 1.32  (1.02, 1.72)* | n/a | n/a | n/a | n/a | n/a | n/a | n/a |
| No reading newspaper (Ref: Yes) | n/a | 1.56  (1.04, 2.34)* | n/a | n/a | n/a | n/a | 1.50 (1.00, 2.25)* | 1.53  (1.13, 1.94)* | n/a |
| ***Child Characteristics*** |  |  |  |  |  |  |  |  |  |
| Child age 6-11 mo (Ref: 12-23 mo) | 1.62  (1.03, 2.54)* | 2.05  (1.59, 2.65)* | 1.79  (1.46, 2.19)* | 1.50  (1.20, 1.86)* | 2.32  (1.49, 3.63)* | 1.34  (1.04, 1.72)* | 1.20  (1.01, 1.43)* | 1.38 (1.07, 1.79)* | 2.56 (1.80, 3.68)* |
| Birth order 1-4th (Ref: 1st) | n/a | 0.89  (0.63, 1.27) | 1.33  (1.01, 1.75)* | 1.05  (0.80, 1.38) | n/a | 1.02  (0.75, 1.39) | n/a | n/a | n/a |
| Birth order 5th or lower (Ref: 1st) | n/a | 1.18  (0.70, 1.98) | 1.98  (1.31, 3.00)* | 0.71 (0.52, 0.98)* | n/a | 1.04  (0.66, 1.63) | n/a | n/a | n/a |
| No vitamin-A supplement (Ref: Yes) | n/a | 1.46 (1.11, 1.93)* | 1.17  (0.97, 1.40) | 1.76  (1.32, 2.34)* | n/a | 1.14 (0.90, 1.45) | 1.19  (0.99, 1.43) | 1.58  (1.22, 2.06)* | 1.03 (0.70, 1.56) |
| Cough (Ref: no) | n/a | n/a | 1.31  (1.06, 1.63)* | n/a | n/a | n/a | 1.16  (0.98, 1.38) | n/a | n/a |
| Diarrhea (Ref: no) | n/a | n/a | n/a | n/a | n/a | n/a | n/a | n/a | n/a |
| Fever (Ref: no) | n/a | n/a | 1.18  (0.96, 1.44) | n/a | n/a | n/a | n/a | n/a | n/a |
| ***Community Characteristics*** |  |  |  |  |  |  |  |  |  |
| Mid tertile of % primary education completion (Ref: low tertile) | 0.79  (0.41, 1.50) | 1.12  (0.78, 1.61) | 0.82  (0.63, 1.07) | 1.04  (0.79, 1.37) | n/a | 0.95  (0.67, 1.36) | 0.80  (0.62, 1.03) | 0.85  (0.62, 1.17) | 1.04  (0.70, 1.56) |
| High tertile of % primary education completion (Ref: low tertile) | 0.90  (0.45, 1.82) | 0.93  (0.63, 1.39) | 1.03  (0.76, 1.39) | 1.12  (0.84, 1.48) | n/a | 0.77  (0.52, 1.13) | 0.95  (0.70. 1.29) | 0.76  (0.52, 1.11) | n/a |
| Mid tertile of % facility delivery (Ref: low tertile) | 1.02  (0.59, 1.80) | 1.15  (0.78, 1.67) | 1.09  (0.84, 1.42) | n/a | n/a | 0.85  (0.59, 1.23) | 1.14  (0.90, 1.44) | n/a | n/a |
| High tertile of % facility delivery (Ref: low tertile) | 0.97  (0.47, 2.02) | 0.88  (0.56, 1.38) | 0.98  (0.74, 1.29) | n/a | n/a | 0.82  (0.53, 1.25) | 1.02  (0.76, 1.37) | n/a | n/a |
| Mid tertile of % % women empowerment (Ref: low tertile) | 1.02  (0.59, 1.80) | 1.11  (0.81, 1.53) | 1.09  (0.84, 1.42) | n/a | n/a | 0.85  (0.59, 1.23) | 1.14  (0.90, 1.44) | n/a | 0.90  (0.62, 1.31) |
| High tertile of % women empowerment (Ref: low tertile) | 0.97  (0.47, 2.02) | 0.92  (0.65, 1.29) | 0.98  (0.74, 1.29) | n/a | n/a | 0.82  (0.53, 1.25) | 1.02  (0.76, 1.37) | n/a | n/a |
| multicollinearity |  |  |  |  |  |  |  |  |  |
| Variance inflation factor | 1.08 | 1.23 | 1.11 | 1.14 | 1.11 | 1.04 | 1.07 | 1.07 | 1.11 |
| Tolerance | 0.92 | 0.81 | 0.90 | 0.88 | 0.90 | 0.96 | 0.96 | 0.94 | 0.90 |

^1^ All values are estimated accounted for survey design and sampling weights.

^2^ *p<0.05

**Supplemental Table 6**. Introduction of solid, semi-solid or soft foods among infants 6–8 months of age among nine countries

| Characteristics | Ethiopia | Kenya (n=500) | Malawi | Rwanda | South Africa (n=872) | Tanzania | Uganda | Zambia | Zimbabwe |
| --- | --- | --- | --- | --- | --- | --- | --- | --- | --- |
|  | (n=545) |  | (n=786) | (n=456) |  | (n=533) | (n=781) | (n=646) | (n=1656) |
|  | ^Weighted %^ | ^Weighted %^ | ^Weighted %^ | ^Weighted %^ | ^Weighted %^ | ^Weighted %^ | ^Weighted %^ | ^Weighted %^ | ^Weighted %^ |
| Rural | 58.1 | 77.1 | 83.5 | 56.7 | 78.5 | 91.4 | 80.8 | 82.9 | 84.5 |
| Urban | 73.9 | 85.8 | 92.7 | 56.1 | 72.6 | 94.2 | 79.8 | 85.9 | 87.4 |
| p | 0.02 | 0.03 | 0.03 | 0.92 | 0.50 | 0.49 | 0.83 | 0.42 | 0.68 |
| 1st quintile | 53.0 | 68.9 | 83.4 | 51.0 | 74.4 | 88.5 | 75.4 | 84.8 | 82.8 |
| 5th quintile | 68.1 | 84.4 | 87.7 | 61.0 | 81.0 | 95.5 | 85.0 | 89.4 | 95.8 |
| p | 0.66 | 0.01 | 0.65 | 0.48 | 0.98 | 0.31 | 0.42 | 0.29 | 0.19 |

^1^ Proportion of infants 6–8 months of age who receive solid, semi-solid or soft foods

^2^ All values (95% CI) are estimated accounted for survey design and sampling weights.
